# Age‐Associated Dysregulation of Postsynaptic Mitochondria Perturbs Reinnervation Kinetics

**DOI:** 10.1111/acel.70355

**Published:** 2026-01-06

**Authors:** Steve D. Guzman, Paula M. Fraczek, Klimentini Itsani, Esraa K. Furati, Devin Juros, Grace Kenney, Gregorio Valdez, Joe V. Chakkalakal, Carlos A. Aguilar

**Affiliations:** ^1^ Department of Biomedical Engineering University of Michigan Ann Arbor Michigan USA; ^2^ Biointerfaces Institute University of Michigan Ann Arbor Michigan USA; ^3^ Department of Orthopaedic Surgery and Cell Biology Duke University Durham North Carolina USA; ^4^ Department of Molecular Biology, Cell Biology and Biochemistry Brown University Providence Rhode Island USA; ^5^ Center for Translational Neuroscience, Robert J. and Nancy D. Carney Institute for Brain Science Brown University Providence Rhode Island USA; ^6^ Center on the Biology of Aging Brown University Providence Rhode Island USA; ^7^ Program in Cellular and Molecular Biology University of Michigan Ann Arbor Michigan USA

## Abstract

Age‐associated degeneration of neuromuscular junctions (NMJs) contributes to sarcopenia and motor function decline, yet the mechanisms that drive this dysfunction in aging remain poorly defined. Here, we demonstrate that postsynaptic mitochondria are significantly diminished in quantity in old‐aged skeletal muscle, correlating with increased denervation and delayed reinnervation following nerve injury. Single‐nucleus RNA sequencing before and after sciatic nerve crush from young and old‐aged muscles further revealed that sub‐synaptic myonuclei in old‐aged muscle exhibit attenuated expression of mitochondrial gene programs, including oxidative phosphorylation, biogenesis, and import. To test whether these deficits are causal, we developed a muscle‐specific CRISPR genome editing approach and targeted CHCHD2 and CHCHD10—two nuclear‐encoded mitochondrial proteins that localize to the intermembrane space and interact with the mitochondrial contact site and cristae organizing system. CRISPR knockout of CHCHD2 and CHCHD10 in young muscle recapitulated old‐aged muscle phenotypes, including mitochondrial disorganization, reduced ATP production, NMJ fragmentation, and delayed reinnervation. Transcriptional profiling of sub‐synaptic myonuclei using single‐nuclei RNA sequencing from CHCHD2 and CHCHD10 knockout muscles revealed impairments in activation of mitochondrial remodeling programs and elevated stress signatures when compared with controls. These findings establish a critical role for postsynaptic mitochondrial integrity in sustaining NMJ stability and regenerative capacity and identify CHCH domain‐containing proteins as key regulators of postsynaptic mitochondrial function during aging and injury.

## Graphical abstract

We show that aging results in postsynaptic mitochondrial loss at the neuromuscular junction and impaired muscle reinnervation. In vivo CRISPR knockout of mitochondrial genes, CHCHD10 and CHCHD2, in young muscles led to fragmented endplates, aberrant innervation, and impaired transcriptional maturation of sub‐synaptic myonuclei during regeneration.

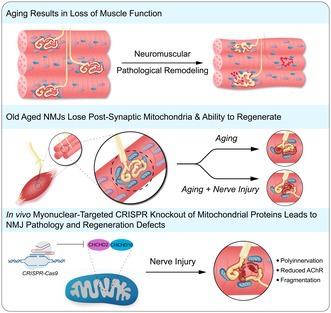

## 1 Introduction

The elderly population (> 60 years) (Lutz et al. 2008) has dramatically increased in number over the last four decades, and this growth has engendered new challenges for healthcare (Hung et al. 2011). A significant contributor to age‐related pathologies is sarcopenia, or age‐associated loss of muscle mass and strength (Marcell 2003). A key determinant of sarcopenia initiation and progression is a reduction in synaptic integrity at neuromuscular junctions (NMJs) (Ham et al. 2020; Jang and Van Remmen 2011). The NMJ is a critical structure within the motor unit and enables the stimulation of muscle fibers by their respective neurons, resulting in excitation‐contraction coupling and force generation. In old age, loss of acetylcholine receptors (AChRs) and NMJ degeneration contribute to the accumulation of chronically denervated muscle fibers (Li et al. 2018), which can cause atrophy and reduce muscle strength and coordination. NMJ degeneration during aging has been shown (Martineau et al. 2018) to be the result of imbalances between denervation and reinnervation (Li et al. 2011), and previous research has also shown that old‐aged axons can regenerate efficiently (Kang and Lichtman 2013) and reach motor endplates after NMJ injury (Sakuma et al. 2016). These results implicate postsynaptic impairments (Li et al. 2011) to promote reinnervation with old age (Taetzsch and Valdez 2018; Aare et al. 2016), but the cellular and molecular mechanisms that promote reinnervation and deleterious change in old age remain underexplored. Currently, there are no therapies to restore motor function for sarcopenic patients (Li et al. 2018). Accordingly, it is critical to elucidate factors that preserve the integrity and function of NMJs to prevent denervation in old age.

Mitochondria have long been recognized as crucial for synaptic maintenance and are enriched at both the pre‐ and post‐synapse (Vos et al. 2010). Mitochondria generate the ATP required to transmit action potentials, regulate reactive oxygen species (ROS) levels, buffer Ca^2+^ ions, and generate acetyl‐CoA, which serves as a precursor for acetylcholine (Anagnostou and Hepple 2020; O'Connor et al. 2023). The organization of the mitochondrial network in myofibers has been linked to denervation‐induced muscle atrophy, whereby healthy muscle mitochondria typically exhibit long and fused networks. However, after denervation, muscle mitochondria display increased fission and autophagy (Romanello et al. 2010). Perturbations to muscle mitochondrial signaling through overactivation of mTORC1 (Ham et al. 2020) or loss of the antioxidant enzyme superoxide dismutase 1 (Jang et al. 2010) (SOD1) induces sarcopenia‐like features in NMJs such as denervation, fragmentation of AChRs, and loss of muscle function. Additionally, various neurogenerative diseases (e.g., Parkinson's, Alzheimer's, and Huntington's disease) and aging (Van Remmen and Richardson 2001) display a dysregulation of mitochondrial fission and fusion dynamics (Chen and Chan 2009). Whether changes in postsynaptic mitochondria in old age are a cause or consequence of NMJ degeneration remains to be established.

Herein, we quantified postsynaptic mitochondria at AChRs from young and old‐aged murine muscles and observed a stark loss of mitochondria in old age that was associated with denervation, AChR fragmentation, and polyinnervation. We next used single‐nuclei RNA sequencing before and after regenerative nervous injury from young and old‐aged muscles and quantified changes in expression of multiple cell types, including sub‐synaptic myonuclei. Analysis of sub‐synaptic myonuclei revealed age‐dependent differences, with sub‐synaptic nuclei from young muscle exhibiting enhanced expression of genes involved in mitochondrial function and oxidative phosphorylation when compared with old‐aged sub‐synaptic nuclei. To probe the functional relevance of these mitochondrial changes in old age, we developed a myonucleus‐specific genome editing system to target mitochondrial regulators. Using this system, we knocked out CHCHD2 and CHCHD10, two proteins associated with the mitochondrial contact site and cristae organizing system (MICOS). The acute loss of CHCHD2 and CHCHD10 proteins in skeletal muscle impaired mitochondrial function and compromised the NMJ's adaptive response to denervation. Together, our data and tools provide new insight into the role of postsynaptic mitochondria in maintaining NMJ integrity throughout aging.

## 2 Results

### 2.1 Mitochondrial Number at the NMJ is Significantly Diminished in Old‐Aged Skeletal Muscle

**FIGURE 1:**
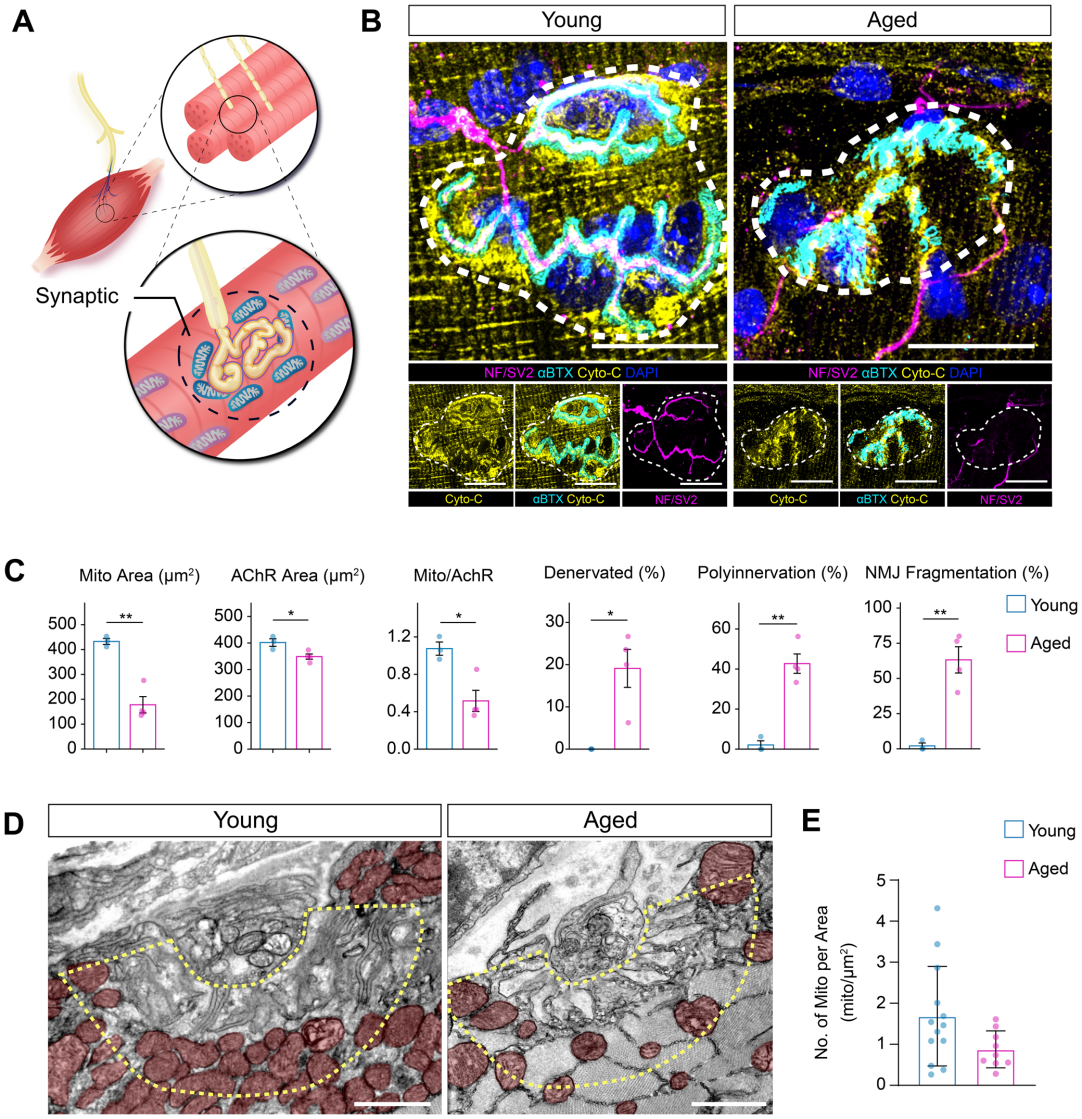
NMJ‐associated mitochondria are enriched in young muscle fibers compared with aged. (A) Schematic depicting the neuromuscular junction (NMJ) and the localization of postsynaptic mitochondria within the muscle fiber. (B) Representative Airyscan2 confocal images of NMJs from young (3‐month‐old) and aged (27‐month‐old) gastrocnemius (GTN) muscles, stained for mitochondria (Cytochrome‐C), acetylcholine receptors (α‐Bungarotoxin, BTX), motor neuron axons (neurofilament, NF), axon terminals (synaptic vesicle protein 2, SV2), and nuclei (DAPI). The white dashed circle represents the NMJ region. (C) Quantification of NMJ‐associated mitochondrial features and NMJ morphology. (D) Transmission electron microscopy (TEM) images of postsynaptic regions from young and aged muscles showing mitochondria (false‐colored red) adjacent to the synaptic membrane (yellow dashed line). (E) Quantification of mitochondrial number per postsynaptic area (mito/μm^2^) from TEM images. Bars represent mean ± SD. *p < 0.05, **p < 0.01 by unpaired two‐tailed Student's *t*‐test. Scale bars (B and D) = 20 μm and 1 μm, respectively.

Age‐related degeneration of the NMJ and motor dysfunction have been shown to drive muscle atrophy, but how changes in mitochondria at the endplate change in old age requires further investigation. To evaluate how skeletal muscle mitochondria are altered at NMJs in old age, we performed immunostaining for presynaptic nerve terminals (neurofilament‐NF and synaptic vesicle 2‐SV2), postsynaptic AChRs (α‐bungarotoxin or α‐BTX), and postsynaptic mitochondria (cytochrome‐C) on myobundles from young (3–4 months) and old‐aged (28 months) tibialis anterior muscles (Figure 1A; *n* = 3–4 per age group). Old‐aged NMJs displayed less total mitochondrial area at NMJs and less overlap with AChRs when compared with young muscles (Figures 1B,C and S1A). The loss of total mitochondria at NMJs also correlated with reductions in AChR size as well as increases in denervation (overlap between pre‐ and postsynaptic compartments), polyinnervation, and NMJ fragmentation (Figure 1B,C), which is consistent with previous studies of old‐aged NMJs (Valdez et al. 2010).

To understand if the loss of mitochondria also coincided with diminished mitochondrial function in old‐aged muscle, we performed a Seahorse XF assay on isolated mitochondria from young (*n* = 3 months) and old‐aged (22 months) skeletal muscles (Figure S1B). We found that young mitochondria exhibited greater overall oxygen consumption rate (OCR) throughout the assay, suggesting increased activity of the oxidative phosphorylation pathway (Figure S1B). We also detected that young mitochondria had higher rates of ATP production and increased maximal respiration when compared with old‐aged mitochondria (Figure S1C,D). These findings agree with previous reports of mitochondrial energy production through oxidative phosphorylation decreasing in old‐aged muscle (Seo et al. 2016; Lesnefsky and Hoppel 2006).

To further validate mitochondrial loss at NMJs in old age, we performed transmission electron microscopy (TEM) on NMJs from young (3 months) and old‐aged (29 months) muscles (Figure 1D). TEM imaging revealed that old‐aged NMJs contained visibly fewer mitochondria within the postsynaptic region compared with young NMJs (Figure 1D), mirroring the decrease in cytochrome c signal detected by confocal imaging. Quantification of mitochondrial number per postsynaptic area showed a downward trend in old‐aged muscles relative to young (Figure 1E), consistent with reduced mitochondrial abundance at the NMJ. Together, these findings demonstrate a loss of postsynaptic mitochondrial content near the endplate in old‐aged skeletal muscle, which may contribute to diminished local bioenergetic capacity and impaired synaptic maintenance.

### 2.2 Old‐Aged NMJs Display Attenuated Ability to Reinnervate After Nerve Injury

NMJ degeneration in old age has been shown to be the result of imbalances between denervation and reinnervation (Li et al. 2011; Aare et al. 2016) To examine changes in the kinetics of denervation and reinnervation with old age, we employed sciatic nerve crush injury (SNCr) on young (4 months) and old‐aged (24 months) tibialis anterior muscles (Liu and Chakkalakal 2018) and immunostained NMJs for pre‐ and postsynaptic compartments as above (Figure S1E). At 7 days post injury (dpi), all young and old‐aged NMJs were observed to be denervated (Figure S1F). At 28 dpi, 89% of young compared with only 28% of old‐aged NMJs were reinnervated (Figure S1F). Although reinnervation increased with longer periods of recovery for young muscles, a significant fraction of old‐aged NMJs (25%) remained denervated at 56 dpi (Figure S1F). These results of delayed NMJ reinnervation and persistent denervated state among a subset of old‐aged NMJs are consistent with previous studies (Li et al. 2011; Aare et al. 2016; Wang et al. 2007) and suggest an association between mitochondrial loss and dysfunction, and reduced ability to reinnervate.

### 2.3 Single Nucleus RNA‐Sequencing of Young and Old‐Aged Muscle After Sciatic Nerve Crush Reveals Alterations to Postsynaptic Response in Old Age

**FIGURE 2:**
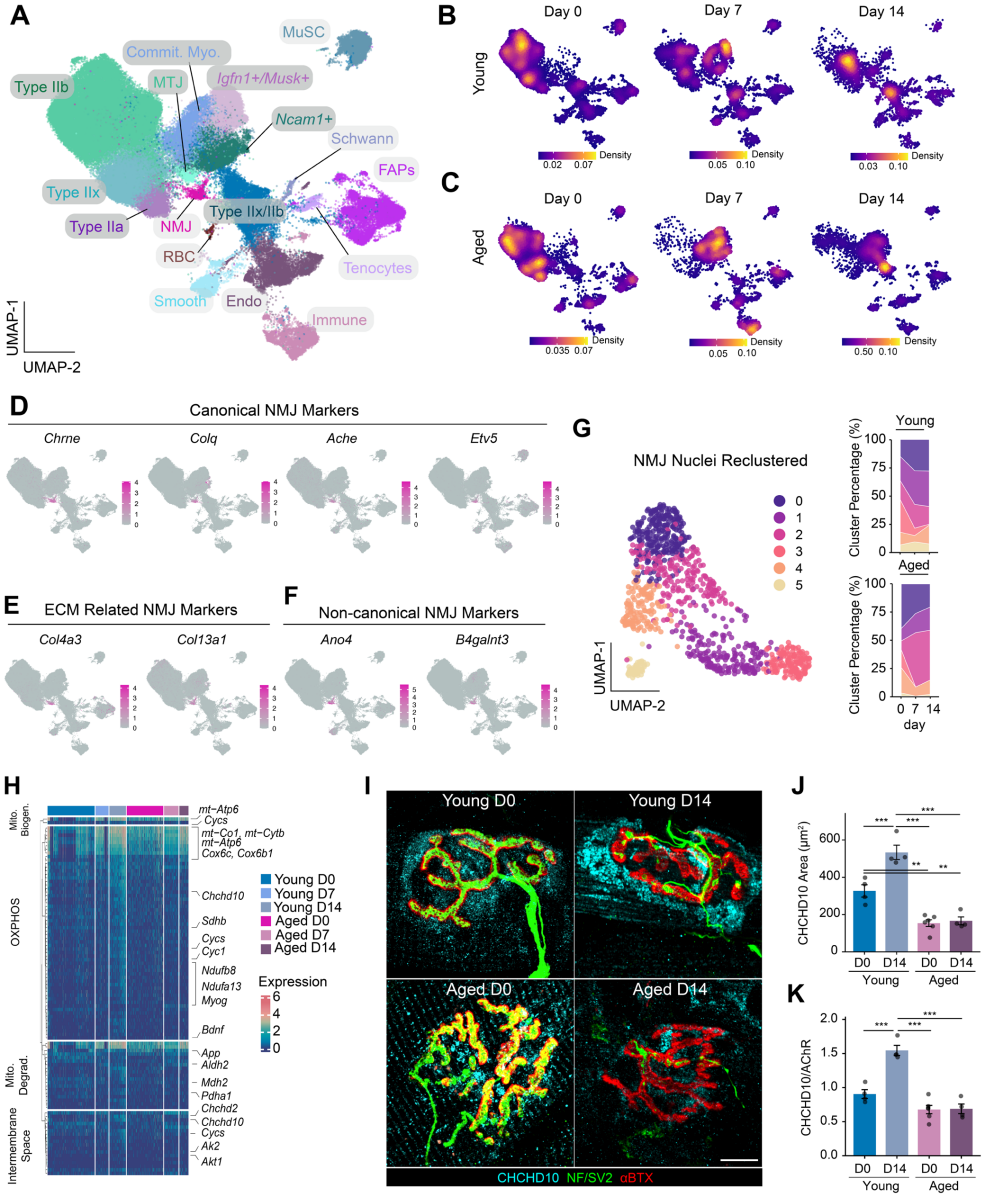
Age‐related transcriptional differences of mitochondrial genes in neuromuscular junction (NMJ)‐associated myonuclei following nerve injury. (A) UMAP projection of single‐nucleus RNA‐seq (snRNA‐seq) data from tibialis anterior muscles across age and time points post‐injury (*n* = 119,782 nuclei). Louvain clustering identified 17 major clusters, including sub‐synaptic myonuclei (NMJ), immune cells, fibro‐adipogenic progenitors (FAPs), and myonuclei of various fiber types. (B and C) Density plots of nuclei distributions across time points in young (B) and old (C) muscles reveal age‐specific shifts in NMJ‐associated myonuclei. (D) Feature plots showing expression of canonical NMJ markers (*Chrne*, *Colq*, *Ache*, *Etv5*) in the NMJ cluster. (E) Expression of extracellular matrix‐related NMJ transcripts (*Col4a3*, *Col13a1*). (F) Noncanonical NMJ‐associated markers (*Ano4*, *B4galnt3*) recently identified as enriched in sub‐synaptic myonuclei. (G) Reclustering of NMJ‐associated nuclei reveals five transcriptionally distinct clusters, with cluster 5 enriched in young muscle and reduced in aged muscle. (H) Heatmap of mitochondrial gene modules shows time‐dependent upregulation of oxidative phosphorylation (OXPHOS), mitochondrial biogenesis, import, and intermembrane space‐associated transcripts in NMJ myonuclei from young but not aged muscle after injury. (I) Representative Airyscan2 confocal images of NMJs from young and aged muscles at baseline (Day 0) and 14 days post‐injury, stained for CHCHD10 (cyan), presynaptic markers NF/SV2 (green), and postsynaptic acetylcholine receptors labeled with α‐bungarotoxin (α‐BTX, red). (J) Quantification of CHCHD10‐positive area at NMJs across age and time points. (K) Quantification of the CHCHD10/AChR ratio showing significantly greater mitochondrial enrichment at NMJs of young muscles at 14 dpi compared with aged muscles. Bars represent mean ± SEM (*n* = 4–6 per group). Statistical significance determined by one‐way ANOVA with Tukey post hoc test. Asterisks indicate significance levels: **p < 0.01, ***p < 0.001. Scale bar = 10 μm.

To elucidate the molecular mechanisms of the postsynaptic response to nerve injury across the lifespan, we performed single‐nucleus RNA sequencing (Petrany et al. 2020; Kim et al. 2020) (snRNA‐Seq) on *tibialis anterior* (TA) muscles isolated from young (3 months) and old‐aged (23–28 months) mice 0, 7, and 14 dpi following SNCr (Figure 2). We generated 119,782 high‐quality snRNA‐Seq libraries (42,675 from young 0 dpi, 19,797 from 7 dpi, 7256 from 14 dpi, 28,273 from old‐aged 0 dpi, 13,975 from old‐aged 7 dpi, and 7806 from old‐aged 14 dpi) encompassing, on average, 2070 genes and 6992 unique molecular identifiers (UMIs) per nucleus (Figure S2A). Ambient RNA was removed using DecontX (Yang et al. 2020) and technical variations between batches were regressed out using scVI Tools (Gayoso et al. 2022) followed by dimensionality reduction using Uniform–Manifold Approximation and Projection (Becht et al. 2019) (UMAP) (Figure 2A). Unsupervised Louvain clustering revealed 17 cell types (Figure 2A), which were annotated by matching unique marker genes with previously published datasets (Kim et al. 2020; Yang et al. 2020, 2023; Giordani et al. 2019) (Figure S2B). We observed recovery of both myonuclei and non‐myonuclei (Figure S2C) and observed similar nuclei to previously published snRNA‐Seq datasets (Lin et al. 2022), including a *Chrne* + myonuclei cluster corresponding to sub‐synaptic myonuclei (labeled as “NMJ”), and an *Igfn1*+ myonuclei cluster, which has been previously observed in denervated muscle and is associated with an atrophic signature (Kim et al. 2020; Xiang et al. 2021) (Figure 2A). We also detected myonuclei expressing Collagen 22 alpha 1 (*Col22α1*), which have been associated with the myotendinous junction (MTJ) as well as different muscle fiber types, including Type II A (*Myh2*+), Type IIB (*Myh4*+), and Type IIX (*Myh1*+).

In young mice, we found that SNCr induced an increase in *Igfn1*+, a marker of atrophy, and *Musk +* myonuclei at 7 dpi that did not cluster with NMJ myonuclei and was reduced by 14 days (Figure S2C). In old‐aged mice, we detected higher proportions of *Igfn1*+ and *Musk* + myonuclei at 7 and 14 dpi compared with young (Figure S2C), potentially suggesting higher degrees of tissue atrophy and response to denervation. Old‐aged muscle also displayed a significant increase in immune cells at 7 dpi compared with young mice (Figure S2C), suggesting greater levels of inflammation (Xiang et al. 2021). We also observed a loss of fast‐twitch myofiber nuclei (Type IIb and Type IIx) following SNCr in young and old‐aged muscles (Figure S2C), which agrees with previous work showing that fast‐twitch fibers are more susceptible to denervation and atrophy (Coletti et al. 2022; Miljkovic et al. 2015). We also detected increased recovery of mixed Type IIx/Type IIb myonuclei after injury and more mixed myonuclei at 14 dpi in old‐aged muscles, which is consistent with human aging that also displayed increases in mixed myofiber types with old age (Lai et al. 2024; Kedlian et al. 2024). These mixed myonuclei also exhibited elevated expression of genes associated with mitochondrial function, including pathways related to aerobic respiration and oxidative phosphorylation (Figure S2D,E). This mitochondrial transcriptional induction at 14 dpi aligns with the timing of reinnervation (Bermedo‐Garcia et al. 2022), and may reflect the reactivation of metabolic programs that accompany muscle recovery (Li et al. 2022).

To evaluate how old age influences transcriptional states within myonuclei that exhibited changes in response to SCNr, we compared gene expression between young and old‐aged muscles at 14 dpi in *Igfn1* ^+^ and *Ncam* ^+^ myonuclear populations (Figure S2F,G). In both *Igfn1* ^+^ and *Ncam* ^+^ young populations, we observed robust enrichment of transcripts including Neurobeachin (*Nbea*), receptor tyrosine kinase‐like orphan receptor 1 (*Ror1*), and Niban Apoptosis Regulator 1 (*Niban1*), each of which plays a role in pathways relevant to nervous regeneration and stress adaptation. *Nbea* is a scaffolding protein required for synaptic transmission and vesicle trafficking at the neuromuscular junction and has also been implicated in maintaining postsynaptic integrity in neurons (Martin et al. 2023). *Ror1* has been shown to modulate neurite growth (Paganoni and Ferreira 2005) and in the central nervous system, and proper reinnervation (Diaz‐Horta et al. 2016). Lastly, NIBAN1, a stress‐inducible protein, has been shown to modulate apoptosis and protein homeostasis, especially under conditions of cellular stress (Diana and Carvalheira 2022), which may be particularly relevant in old‐aged myonuclei. Together, these findings suggest that young myonuclei respond to denervation through activation of genes associated with synaptic stability, reinnervation, and stress resilience, while old‐aged myonuclei show a blunted or incomplete activation of these gene programs during regeneration.

The rarity of sub‐synaptic myonuclei in myofibers has made it difficult to study their transcriptional programs and response to injury, such as denervation. The sensitivity of our approach enabled recovery of sub‐synaptic myonuclei before and after SNCr in young and old age (Figure 2D). We recovered myonuclei that specifically expressed synaptic genes, such as *Chrne, Ache*, *and Etv5* (Figure 2D). We also detected sub‐synaptic myonuclei that specifically expressed transcripts that are associated with synaptic basal lamina, such as collagen Q (*Colq*), collagen 4 alpha 3 (*Col4α3*), and collagen 13 alpha 1 (*Col13α1*), and noncanonical markers, anoctamin 4 (*Ano4*) and Beta‐1,4‐N‐Acetyl‐Galactosaminyltransferase 3 (*B4galnt3*). *Ano4* encodes a calcium‐activated chloride channel predominantly expressed in the hypothalamus, where it plays a role in glucose‐sensing (Sjöstedt et al. 2020), and *B4galnt3* encodes a glycosyltransferase involved in the synthesis of specific glycan structures (Hsu et al. 2011). The detection of these markers in sub‐synaptic myonuclei suggests potential roles in modulating synaptic function and metabolic responses at the NMJ (Figure 2D–F).

We reclustered the sub‐synaptic myonuclei and identified five distinct clusters (Figure 2G). Gene Ontology (GO) enrichment analysis revealed that Cluster 1 was enriched for terms related to the neuromuscular junction (NMJ), including postsynaptic specialization and postsynaptic membrane. Clusters 2 through 4 were enriched for terms associated with the plasma membrane, synapse‐associated extracellular matrix, sarcoplasm, and growth cone (Figure S3A). We focused on Cluster 5, which exhibited consistently higher representation in young muscles compared with old‐aged muscles at all time points (Figure 2G). GO analysis of Cluster 5 revealed significant enrichment for mitochondrial‐related pathways (Figure S3A), and similar mitochondrial terms—such as “mitochondrial respirasome” and “inner mitochondrial membrane protein complex”—were among the top five pathways enriched in NMJ nuclei from young versus old‐aged muscles at 14 dpi, based on gene set enrichment analysis (Subramanian et al. 2005) (Figure S3B). To further evaluate age‐related differences in mitochondrial gene programs, we scored genes involved in specific mitochondrial pathways—such as oxidative phosphorylation, mitochondrial biogenesis, import, and degradation—using curated gene modules (see Table 1 for gene lists). Young NMJ myonuclei displayed robust, time‐dependent upregulation of these mitochondrial modules following injury, whereas old‐aged sub‐synaptic myonuclei showed minimal activation across the same pathways (Figure S3C). We were particularly interested in genes involved in multiple mitochondrial processes, and detected two genes coiled–coil–helix–coiled–coil–helix (Modjtahed et al. 2016) (CHCH) 10 (*Chchd10*) and cytochrome C (*Cycs*), which were components of more than one module (e.g., oxidative phosphorylation, mitochondrial degradation, and intermembrane space) and were selectively upregulated in young sub‐synaptic myonuclei after injury, but not in old‐aged sub‐synaptic myonuclei (Figure 2H). Both *Chchd10* (Bannwarth et al. 2014) and *Cycs*, along with *Chchd2* (Baughman et al. 2009), which is a known binding partner of *Chchd10*, were significantly induced in young NMJ nuclei following injury (Figure S3D). CHCHD10 and CHCHD2 are mitochondrial proteins that reside in the intermembrane space and regulate electron flow through the electron transport chain during oxidative phosphorylation (Genin et al. 2016; Aras et al. 2015), and have been linked to multiple neurodegenerative diseases (Kee et al. 2021; Lu et al. 2022). These findings indicate a substantial age‐related impairment in the mitochondrial gene expression program of sub‐synaptic myonuclei, suggesting reduced capacity to maintain and regenerate the postsynaptic compartment following denervation.

**TABLE 1:** Mitochondrial gene modules.

Module name	Gene symbol	Database	Source link
Mitochondrial biogenesis	Atp5pb, Atp5f1d, Atp5mc1, Sod2, Gabpa, Atp5f1e, Atp5mc3, Atp5mj, Glud1, Atp5pf, Atp5po, Atp5f1b, Atp5f1a, Sirt3, Atp5f1c, Gabpb1, Acss2, Sirt4, Idh2, Atp5pd, Atp5mf, Atp5mg, Atp5me, Sirt5, Dmac2l, Gm10053, Atp5mc2, Cycs, mt‐Atp8, mt‐Atp6, Atp5mk	msigdb	https://www.gsea‐msigdb.org/gsea/msigdb/cards/REACTOME_MITOCHONDRIAL_BIOGENESIS
Mitochondrial degradation	Cox5a, Dbt, Spg7, Clpp, Tfam, Prkaca, Ndufs3, Cs, Pdk1, Ndufa2, Clpx, Mrpl32, Slc25a5, Mdh2, Ogdh, Dld, Arg2, Acot2, Acot3, Pdhb, Oxsm, Glud1, Oxct1, Nadk2, Smdt1, Aco2, Atp5pf, App, Atp5po, Ndufv3, Eci1, Hspa9, Afg3l2, Me2, Fech, Aldh18a1, Twnk, Hsd17b10, Atp5f1b, Shmt2, Atp5f1a, Atp5f1c, Ndufs1, Hspd1, Fh1, Iars2, Pmpca, Hmgcs2, Hadh, Aldh2, Ssbp1, Idh2, Acadsb, Uqcrc2, Pdha1, Star, Ldhd, Acad8, Acat1, Idh3a, Pccb, Alas1, Atp5pd, Mrps10, Aldh1b1, Mrps2, Ndufa13, Ndufv1, Atp5mg, Mrpl12, Lonp1, Acot5, Uqcrq, Bdh1, Ech1, Cox5b, Suclg2, mt‐Co1, mt‐Atp6, Htra2, Acot1	msigdb	https://www.gsea‐msigdb.org/gsea/msigdb/mouse/geneset/REACTOME_MITOCHONDRIAL_PROTEIN_DEGRADATION.html
Oxidative phosphorylation	1700066M21Rik, Abcd1, Afg1l, Ak4, Antkmt, Apoc3, Atp5f1a, Atp5f1b, Atp5f1c, Atp5f1d, Atp5f1e, Atp5if1, Atp5me, Atp5mf, Atp5pb, Atp5pd, Atp5pf, Atp5po, Atp6‐ps, Atp7a, Atpsckmt, Bcl2l13, Bdnf, Bid, Ccnb1, Ccnb1‐ps, Cdk1, Chchd2, Chchd2‐ps, Chchd10, Coa6, Coq7, Coq9, Cox4i1, Cox4i2, Cox5a, Cox5b, Cox6a1, Cox6a2, Cox6b1, Cox6b2, Cox6c, Cox7a1, Cox7a2, Cox7a2l, Cox7b, Cox7b2, Cox7c, Cox8a, Cox8b, Cox8c, Cyc1, Cycs, Cyct, Dguok, Dld, Dnajc15, Dnajc30, Fxn, Iscu, Macroh2a1, Mir451a, Mir451b, Mlxipl, Msh2, mt‐Atp6, mt‐Atp8, mt‐Co1, mt‐Co2, mt‐Co3, mt‐Cytb, mt‐Nd1, mt‐Nd2, mt‐Nd3, mt‐Nd4, mt‐Nd4l, mt‐Nd5, mt‐Nd6, Mtch2, Myc, Myog, Ndufa1, Ndufa2, Ndufa3, Ndufa5, Ndufa6, Ndufa7, Ndufa8, Ndufa9, Ndufa10, Ndufa11, Ndufa12, Ndufa13, Ndufab1, Ndufaf1, Ndufb1, Ndufb2, Ndufb3, Ndufb4, Ndufb5, Ndufb6, Ndufb7, Ndufb8, Ndufb9, Ndufb10, Ndufb11, Ndufc1, Ndufc2, Ndufs1, Ndufs2, Ndufs3, Ndufs4, Ndufs5, Ndufs6, Ndufs7, Ndufs8, Ndufv1, Ndufv2, Ndufv3, Nipsnap2, Nupr1, Park7, Pde2a, Pink1, Ppif, Rhoa, Sdha, Sdhaf2, Sdhb, Sdhc, Sdhd, Shmt2, Slc25a23, Slc25a33, Slc25a51, Snca, Stoml2, Tafazzin, Tefm, Tmem135, Tnf, Uqcc2, Uqcc3, Uqcr10, Uqcr11, Uqcrb, Uqcrc1, Uqcrc2, Uqcrfs1, Uqcrh, Uqcrq, Vcp	mgi	https://www.informatics.jax.org/go/term/GO:0006119
Intermembrane space	Agk, Aifm1, Ak2, Akt1, Alpl, Arl2, Arl2bp, AU015836, Bloc1s1, Cdc25c, Cep89, Chchd2, Chchd2‐ps, Chchd4, Chchd5, Chchd7, Chchd10, Ciapin1, Clpb, Cmc4, Coa4, Coa6, Coa7, Cox17, Cox19, Cpox, Cpt1b, Cycs, Cyct, Diablo, Dusp18, Dusp21, Fbxl4, Fgr, Fkbp10, Gatm, Gfer, Ggnbp1, Golph3, Hax1, Hsd3b1, Hsd3b2, Hsd3b3, Hsd3b4, Hsd3b5, Hsd3b6, Hsd3b8, Hsd3b9, Htra2, Immt, Micu1, Micu2, Micu3, Mix23, Myoc, Nbr1, Ndufa8, Ndufb7, Ndufs1, Ndufs5, Nln, Nme4, Oma1, Opa1, Pank2, Park7, Pink1, Pnpt1, Ppox, Prelid1, Prelid2, Prelid3a, Prelid3b, Rexo2, Rnaset2a, Rnaset2b, Sdhaf3, Sirt5, Stmp1, Stoml2, Suox, Them4, Thop1, Timm8a1, Timm9, Timm10, Timm10b, Timm13, Timm23, Timm29, Trap1, Triap1, Uqcc2	mgi	https://www.informatics.jax.org/go/term/GO:0005758

To validate these transcriptomic findings, we performed immunostaining for CHCHD10 alongside presynaptic markers (NF/SV2) and postsynaptic AChRs (α‐BTX) in young (4–5 months) and old‐aged (28–29 months) muscles before and 14 days post SNCr. We detected expression and the area covered by CHCHD10 at NMJs was significantly higher in young muscles at 14 dpi compared with both their uninjured baseline and old‐aged counterparts, consistent with transcriptional upregulation during NMJ remodeling (Figure 2I–K). In contrast, old‐aged NMJs showed minimal CHCHD10 induction following denervation, resulting in markedly reduced CHCHD10 expression and CHCHD10/AChR ratio relative to young injured NMJs (Figure 2I–K). Together, these results demonstrate that aging impairs mitochondrial gene activation and protein recruitment to the postsynaptic region after denervation, revealing a critical deficit in the metabolic resilience of old‐aged NMJs.

### 2.4 Establishment of In Situ Muscle Mitochondria Genome Editing System

**FIGURE 3:**
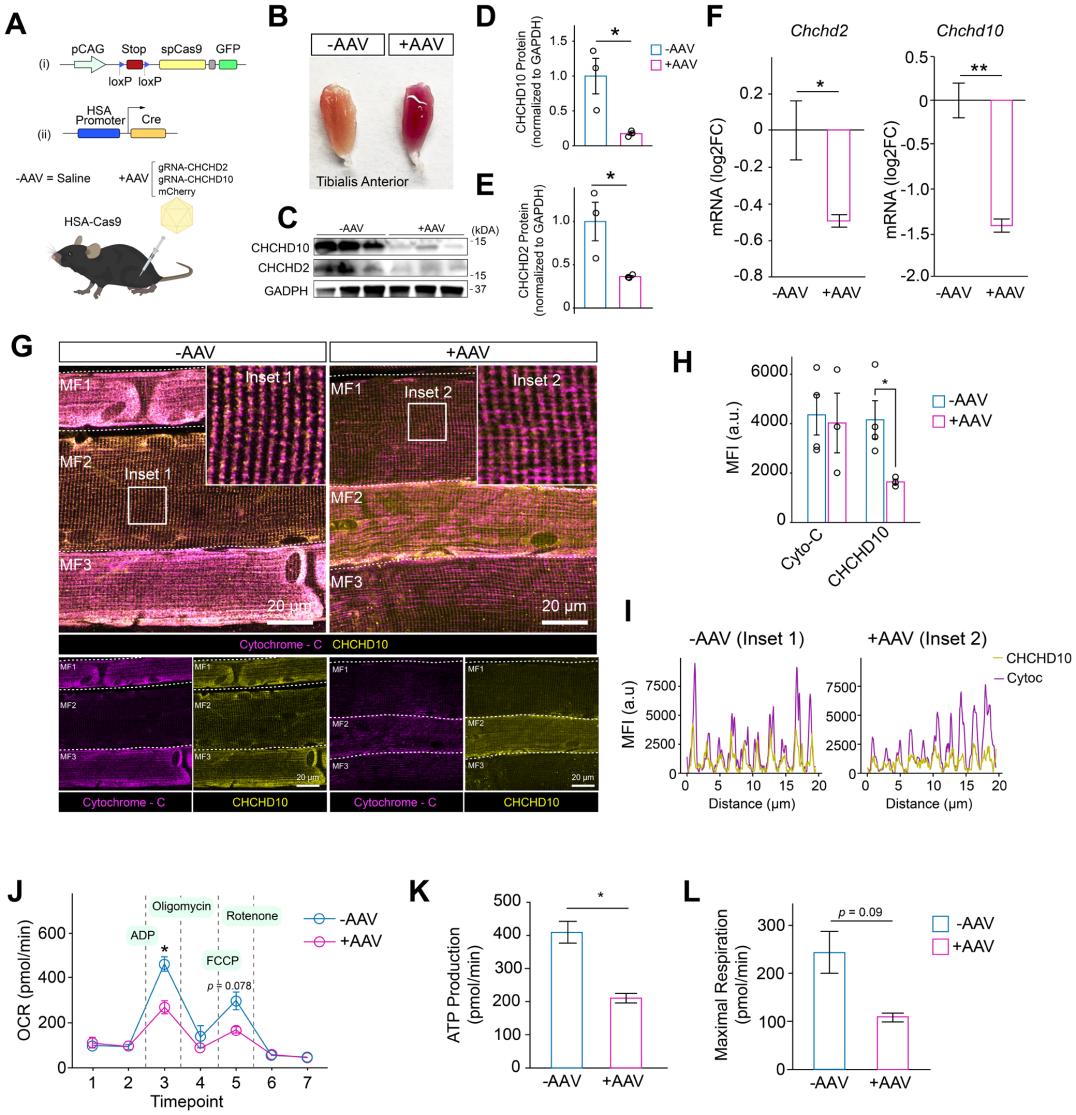
Muscle‐specific knockout of CHCHD2 and CHCHD10 impairs mitochondrial organization and function. (A) Schematic of the muscle‐specific genome editing system. Acta1‐Cre Rosa26^LSL‐Cas9‐eGFP^ mice were injected with AAV9 carrying mCherry and guide RNAs (gRNAs) targeting *Chchd2* and *Chchd10* (+AAV) or saline control (−AAV). (B) Representative tibialis anterior (TA) muscles three weeks after injection showing reduced muscle mass in +AAV compared with −AAV. (C) Western blot analysis of CHCHD2 and CHCHD10 protein expression in −AAV and +AAV TA muscles with GAPDH as a loading control. (D and E) Quantification of CHCHD10 (D) and CHCHD2 (E) protein levels normalized to GAPDH showing significant reduction in +AAV muscles (*n* = 3 per group). (F) RT‐qPCR analysis showing decreased *Chchd2* and *Chchd10* transcript abundance in +AAV muscles compared with −AAV (*n* = 3 per group). (G) Representative confocal images of longitudinal muscle fibers stained for cytochrome c (magenta) and CHCHD10 (yellow) showing reduced signal and disrupted mitochondrial organization in +AAV fibers. Scale bar = 20 μm. Insets highlight regions used for quantification. (H) Quantification of mean fluorescence intensity (MFI) for cytochrome c and CHCHD10 showing decreased mitochondrial signal in +AAV muscles. (I) Line‐scan fluorescence intensity profiles from inset regions in (G) showing loss of coordinated CHCHD10 and cytochrome c localization in +AAV muscles. (J) Oxygen consumption rate (OCR) measured in isolated mitochondria from TA muscles by Seahorse XF analysis. Sequential additions of ADP, oligomycin, FCCP, and rotenone are indicated. (K and L) Quantification of ATP production (K) and maximal respiration (L) showing reduced bioenergetic function in CHCHD2/CHCHD10‐deficient muscles. Bars represent mean ± SEM. Statistical significance was determined by unpaired two‐tailed Student's *t*‐test. Asterisks denote significance levels: *p < 0.05, **p < 0.01.

If impairments in postsynaptic mitochondria attenuate the ability of NMJs to promote reinnervation, we reasoned that knockout of mitochondrial proteins in young muscle would elicit similar dysfunctional responses to reinnervate after nerve injury. To begin to test this, we bred transgenic mice that express *Cre*‐recombinase with human alpha‐skeletal muscle actin (HSA or Acta1‐Cre) with mice that express S. pyogenes CRISPR‐Cas9 and an enhanced green fluorescent protein (eGFP) via the Rosa26 locus (Thurkauf et al. 2023) (Acta1^Cre^–Rosa26^LSL‐Cas9‐eGFP^, Figure 3A). To verify we could edit the genomes of myonuclei in situ, we constructed adeno‐associated virus 9 (AAV9) that contains a fluorescent marker (mCherry) and one short guide RNA (sgRNA) under a U6 promoter targeted toward each of the CHCHD10 and CHCHD2 loci (Figure S4A,B). We administered the AAVs and saline controls through intramuscular injection to the tibialis anterior muscle and allowed mice to recover for 3 weeks. We observed that muscles that received the AAVs exhibited strong mCherry expression compared with muscles that received saline controls (Figures 3B and S4C). We extracted treated and control muscles and performed Western blots for CHCHD10 and CHCHD2 (Figure 3C). We detected significant loss of both CHCHD10 and CHCHD2 for AAV‐treated muscles (Figure 3D,E), which agreed with losses in expression of both genes (Figure 3F). To further confirm decreases in protein levels for AAV‐treated muscles, we extracted myobundles and immunostained for mitochondria (cytochrome‐C) and CHCHD10 (Figure S4D). We detected that the background‐corrected fluorescent intensity of cytochrome‐C was similar for AAV‐treated and control muscles, indicating comparable total levels of mitochondria, but CHCHD10 intensity was significantly attenuated, in agreement with our Western blots (Figure S4E). Additionally, the mitochondrial network was much more disorganized and contained irregular intermyofibrillar mitochondria for AAV‐treated compared with control muscles (Figure S4F). These results are highly similar to observations of muscle mitochondria after denervation (Romanello et al. 2010) and further suggest mitochondrial remodeling in response to CHCHD10 and CHCHD2 knockout.

To glean if CHCHD10 and CHCHD2 knockout induced mitochondrial impairments in function, we extracted mitochondria from AAV‐treated and control muscles and performed Seahorse XF analysis, as described above (Figure 3G). We found that mitochondria from AAV‐treated muscles exhibited lower OCR levels throughout the assay, as well as reductions in ATP production and maximal respiration (Figure 3H,I), similar to the pattern observed in old‐aged mitochondria (Figure 1D–F). These observations of diminished mitochondrial respiration (Straub et al. 2018) and oxidative phosphorylation are consistent with previous studies (Purandare et al. 2018) examining the role of CHCHD10 and CHCHD2 contributions to the electron transport chain. Coupled together with the results above, these results show we can edit myonuclei and multiple mitochondrial genes to mimic old‐aged responses.

### 2.5 Muscle‐Specific Knockout of CHCHD10 and CHCHD2 Promotes Degeneration and Impaired Regeneration of Neuromuscular Synapses

**FIGURE 4:**
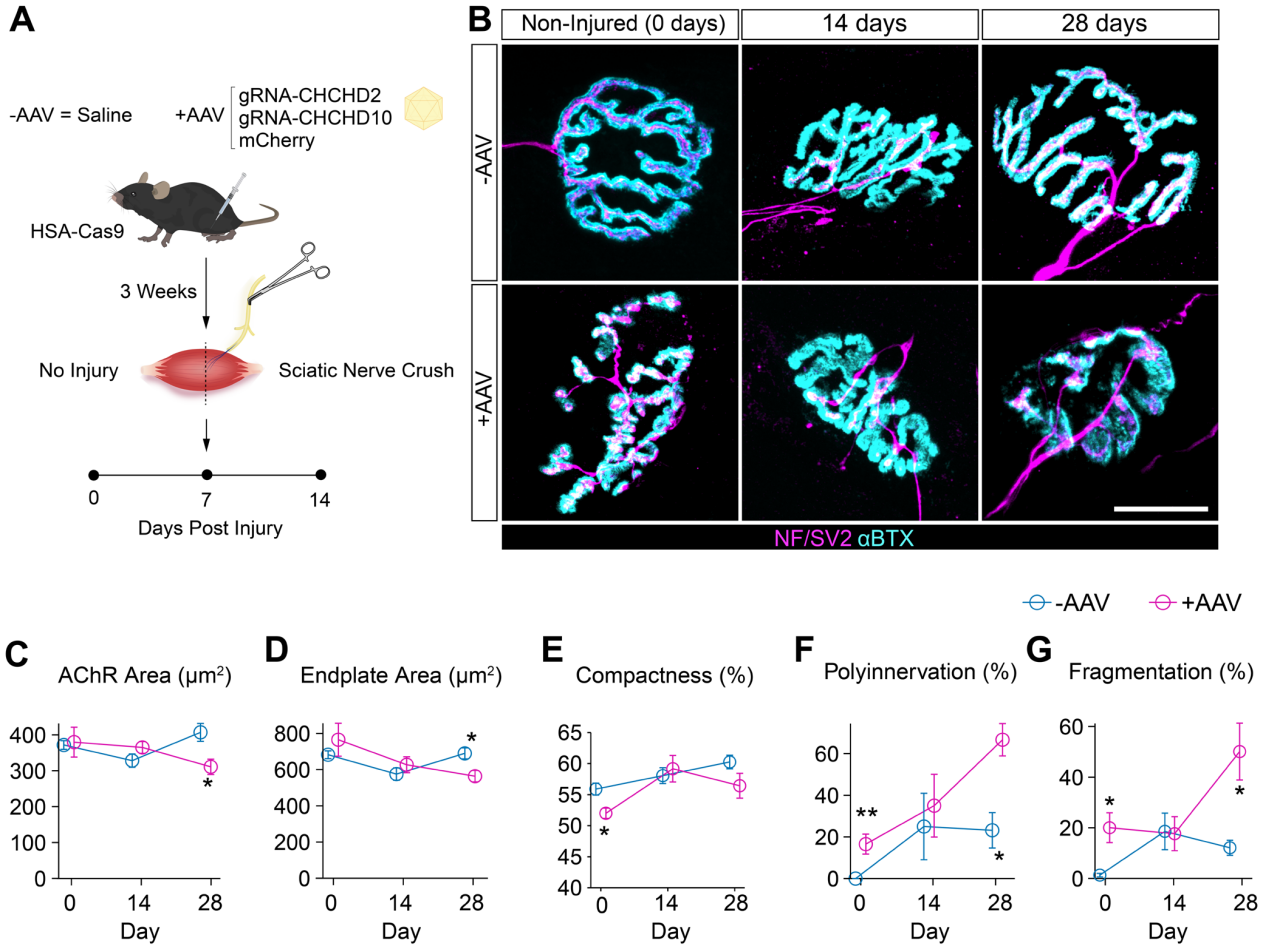
Myonuclear deletion of CHCHD2 and CHCHD10 impairs NMJ structural recovery following nerve injury. (A) Experimental design schematic showing intramuscular injection of AAV encoding *gRNA‐CHCHD2* and *gRNA‐CHCHD10* (mCherry reporter) or saline control (−AAV) into HSA‐Cas9 mice. Animals were subjected to sciatic nerve crush and analyzed at 0, 14, and 28 days post‐injury. (B) Representative confocal images of NMJs from non‐injured, 14‐day, and 28‐day post‐injury muscles stained for presynaptic markers NF/SV2 (magenta) and postsynaptic acetylcholine receptors (α‐bungarotoxin, cyan). Scale bar = 20 μm. (C–G) Quantification of NMJ morphology metrics, including AChR area (C), endplate area (D), compactness (E), polyinnervation (F), and fragmentation (G) in control (−AAV) and CHCHD2/CHCHD10 knockout (+AAV) muscles across time points. +AAV muscles showed reduced endplate size, increased fragmentation, and elevated polyinnervation at 28 days post‐injury, indicating impaired structural regeneration of the NMJ. Data are presented as mean ± SEM (*n* = 3–4 per group). Statistical significance was determined by two‐way ANOVA with Tukey post hoc test. Asterisks denote significance levels: *p < 0.05, **p < 0.01.

To investigate the role of CHCHD10 and CHCHD2 in maintaining NMJ structure, we performed targeted deletion of these genes in myofibers using intramuscular injection of AAVs expressing sgRNAs into HSA‐Cas9 mice, followed by SNCr three weeks later (Figure 4J). We then evaluated NMJ morphology at baseline (day 0), 14, and 28 dpi using immunostaining for pre‐ and postsynaptic compartments (Figure 4K).

At baseline (0 dpi), AAV‐treated NMJs appeared structurally intact, with minimal differences in endplate area, perimeter, or nerve terminal coverage compared with controls (Figure S5). However, even in uninjured muscles, AAV‐treated NMJs exhibited reduced AChR compactness and increased polyinnervation, features typically associated with reinnervation (Figure 4M,N). These findings suggest that CHCHD10/CHCHD2 deletion initiates early structural remodeling at the NMJ in the absence of injury.

Following SNCr, NMJs from AAV‐treated and control muscles exhibited comparable levels of denervation and reinnervation at 14 dpi across most metrics (Figures 4L–O and S5). By 28 dpi, however, AAV‐treated NMJs displayed significant impairments in regeneration, including reduced AChR and endplate area (Figures 4L and S5), decreased postsynaptic compactness (Figure 4M), and sustained elevations in polyinnervation and AChR fragmentation (Figure 4N,O). Together, these results indicate that CHCHD10 and CHCHD2 are required in myofibers to maintain synaptic integrity and support proper NMJ remodeling after injury.

### 2.6 Single Nucleus RNA‐Sequencing Before and After Sciatic Nerve Crush From CHCHD10 and CHCHD2 Knockout and Control Muscles

**FIGURE 5:**
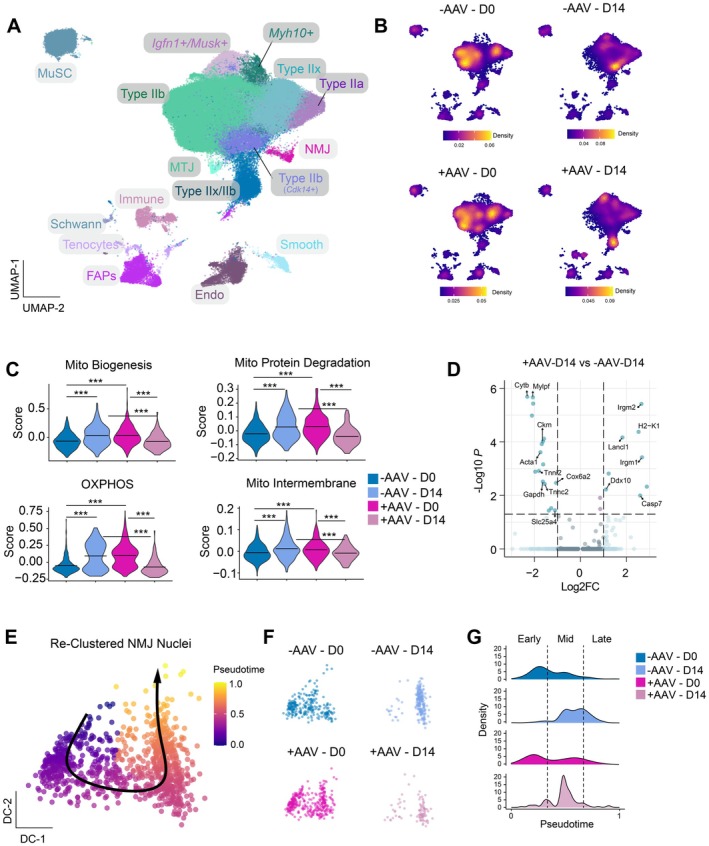
Single‐nucleus RNA sequencing reveals altered NMJ‐associated myonuclear transcriptional states following CHCHD2/CHCHD10 knockout. (A) UMAP projection of single‐nucleus RNA‐seq data from tibialis anterior muscles collected at baseline (Day 0) and 14 days post‐injury (D14) from animals injected with saline (−AAV) or CRISPR‐AAV targeting mitochondrial genes (+AAV). Major myonuclear and non‐myonuclear populations are annotated, including *Igfn1* ^+/^ *Musk* ^+^ denervated myonuclei, *Myh10* ^+^ transitional nuclei, and sub‐synaptic NMJ nuclei. (B) Density plots showing temporal redistribution of NMJ‐associated nuclei in control and AAV‐treated muscles. (C) Violin plots of pathway scores showing significant reductions in mitochondrial biogenesis, oxidative phosphorylation (OXPHOS), protein degradation, and intermembrane‐space gene modules in +AAV samples at both Day 0 and Day 14. (D) Volcano plot comparing +AAV‐D14 vs. −AAV‐D14 NMJ nuclei, highlighting decreased expression of mitochondrial and contractile genes (*Cox6a2, Ckm, Tnni2*) and increased stress‐response transcripts (*Irgm1, H2–K1*). (E) Diffusion‐map–based trajectory of reclustered NMJ nuclei showing pseudotime progression from early to late postsynaptic states. (F) Diffusion maps from each condition highlighting clustering differences and reduced progression toward late‐stage recovery in +AAV samples. (G) Density distribution of pseudotime states demonstrating a shift toward early‐stage transcriptional programs in +AAV‐treated muscles. Bars and violin plots represent mean ± SEM. Statistical significance was determined by one‐way ANOVA with Tukey post hoc test; asterisks denote significance levels (*p < 0.05, **p < 0.01, ***p < 0.001).

To gain insights into the mechanisms that confer defects in NMJs and the ability to promote reinnervation from CHCHD10/2 loss, we performed snRNA‐Seq as above from *tibialis anterior* (TA) muscles at 0 and 14 dpi following SNCr. We generated 131,289 high‐quality snRNA‐Seq libraries with an average of 2184 genes and 7133 UMIs per nucleus and a total of 16 cell clusters (Figure 5A). We recovered highly similar types of nuclei as our young and old‐aged datasets, including myonuclei that express markers of Type IIA, Type IIB, Type IIX myofibers, as well as myonuclei that express both Type IIB and IIX markers (Figures 5B and S6B). We also recovered myonuclei that express *Igfn1*, as well as MTJ and NMJ nuclei (Figure S6B). Additionally, we recovered nuclei from muscle stem cells, endothelial cells, fibro‐adipogenic progenitors, and different types of immune cells (Figure S6B). Prior to injury, we detected highly similar numbers of myonuclei for both AAV+ treated and AAV– control mice (Figure S6C,D). After SNCr, we detected similar increases in Igfn1+ myonuclei for both AAV+ treated and AAV– control mice as for the young and old‐aged datasets responding to injury (Figure S6C,D). We also detected that AAV– control muscles displayed increased recovery of Type IIB myonuclei that expressed *Cdk14*, a canonical Wnt regulator (Chen et al. 2025), which in turn modulates NMJ formation and pre‐patterning (Jing et al. 2009) (Figure S6C,D). Similar to old‐aged mice, AAV+ treated muscles displayed increased recovery of mixed Type IIX/Type IIB myonuclei, as well as larger recovery of immune cells after SNCr, compared with AAV– control muscles (Figure S6C). The elevated proportion of immune cell nuclei in AAV‐treated muscles at 14 days post‐injury suggests that these muscles remain in the degenerative phase of Wallerian degeneration, characterized by ongoing myelin debris clearance and delayed transition to the regenerative phase, thereby potentially impeding timely reinnervation (Gaudet et al. 2011).

To assess the impact of CHCHD2 and CHCHD10 deletion on sub‐synaptic nuclei after SNCr injury, we compared transcriptional profiles of AAV‐treated and control sub‐synaptic nuclei. We performed module scoring for different mitochondrial programs as above and detected oxidative phosphorylation (OXPHOS), mitochondrial biogenesis, protein degradation, and intermembrane space components, all of which showed significantly reduced expression across all modules in AAV‐treated compared with controls (Figure 5C). This result suggests that CHCHD2 and CHCHD10‐deficient sub‐synaptic nuclei ultimately fail to upregulate coordinated mitochondrial programs during reinnervation. We then focused further on differentially expressed genes in sub‐synaptic nuclei at 14 dpi and observed upregulation of immunity‐related GTPase family M members 1 and 2 (*Irgm1* and *Irgm2*), caspase 7 (*Casp7*), and lanthionine synthase C‐like protein 1 (*Lancl1*) for AAV‐treated muscles (Figure 5D). *Irgm1* and *Irgm2* are interferon‐inducible GTPases associated with innate immune responses, mitochondrial integrity, and regulation of autophagy (Rai 2021), suggesting a heightened stress or inflammatory state in CHCHD2 and CHCHD10‐deficient sub‐synaptic nuclei. *Lancl1* is implicated in redox homeostasis (Wang et al. 2018) and neuroprotection (Tan et al. 2020), and may reflect a compensatory antioxidant response to mitochondrial dysfunction. Conversely, control sub‐synaptic nuclei exhibited elevated expression of *Gapdh* and *Ckm*, two mitochondrial genes (Butera et al. 2019; Balberova et al. 2021) associated with energy metabolism and muscle contractility.

To further investigate transcriptional alterations in NMJ nuclei following CHCHD2 and CHCHD10 deletion and during reinnervation, we examined gene expression dynamics along pseudotime trajectories (Figure 5E,F). To define group‐specific transcriptional differences across the pseudotime trajectory, we performed within‐lineage differential gene expression analysis between –AAV and + AAV groups (Figure 5G). In control (–AAV) NMJ nuclei, canonical synaptic genes such as *Gdnf*, *Colq*, and *Kcnq5* exhibited distinct transient peaks at the mid pseudotime states, aligning with their established roles in synaptic maintenance and reinnervation (Figure S6D). Although AAV‐treated nuclei generally retained these temporal expression patterns, their amplitudes were attenuated, suggesting a dampened activation of synaptic gene programs. We also detected that αB‐crystallin (*Cryab*), a small heat shock protein that protects against oxidative stress (Fittipaldi et al. 2015), Insulin‐like Growth Factor Binding Protein 5 (*Igfbp5*), an IGF binding protein that impinges on hypertrophy (Salih et al. 2004), and reticulophagy regulator 1 (*Retreg1 or Fam134b*), an endoplasmic reticulum receptor that promotes degradation by autophagy (Khaminets et al. 2015), were significantly upregulated in AAV‐treated NMJ nuclei, consistent with a sustained stress‐adaptive or injury‐response state observed in old‐aged muscles (Figure S6E). Taken together, these findings indicate that CHCHD2 and CHCHD10 knockout leads to an aberrant transcriptional profile in sub‐synaptic nuclei that fails to sustain the full mitochondrial remodeling response required for effective regeneration.

## 3 Discussion

Degeneration of the neuromuscular synapse is at the center of processes (Gonzalez‐Freire et al. 2014; Chai et al. 2011) related to sarcopenia, and a critical driver of NMJ health and function is the postsynaptic mitochondria. Postsynaptic mitochondria are concentrated at the endplate to provide energy, buffer Ca^2+^, and support synaptic vesicle transport (Ham et al. 2020). We detected that old‐aged NMJs lose postsynaptic mitochondria, and this loss is associated with increased denervation as well as delayed reinnervation after injury. While denervation has been shown to contribute to the fragmentation of mitochondria (Romanello et al. 2010), it has remained an open question whether mitochondrial changes beget NMJ degeneration. To understand how postsynaptic mitochondrial changes contribute to NMJ degeneration and delays in reinnervation kinetics, we utilized a novel muscle‐specific CRISPR editing approach (Platt et al. 2014) to knockout two nuclear‐encoded mitochondrial intermembrane CHCH‐proteins. Mutations in CHCHD2 and CHCHD10 contribute to multiple types of neurodegenerative disorders (Jiang et al. 2022; Ikeda et al. 2022) including amyotrophic lateral sclerosis, Parkinson's disease, and frontotemporal dementia, but have not been observed to be lost in aging. We detected that CRISPR knockout of CHCHD2 and CHCHD10 in young myofibers resulted in increases in polyinnervation and NMJ fragmentation, as well as loss of ability to generate ATP through oxidative phosphorylation and reinnervate after nerve injury, which mimicked old‐aged muscles. We also detected that CHCHD2 and CHCHD10 loss resulted in impaired expression of other mitochondrial genes after nerve injury, suggesting that negative changes in postsynaptic mitochondria predispose NMJs to degeneration and reduce their ability to reinnervate after nerve injury.

Over 1000 of the proteins that make up mitochondria are encoded in the nucleus, and to support the NMJ, these mitochondrial proteins must be expressed by sub‐synaptic myonuclei, translated, and imported into proximal mitochondria. Profiling transcription of mitochondria and synaptic genes from sub‐synaptic myonuclei has been understudied due to the rarity of the nuclei (Larouche et al. 2021; Ham et al. 2025) (< 10 myonuclei in single muscle fibers). The finding that old‐aged sub‐synaptic myonuclei do not increase expression of multiple mitochondrial gene expression programs after nerve injury suggested an impairment in sub‐synaptic myonuclei to recruit axons back and reestablish the NMJ. This result is in line with previous research that has shown old‐aged axons can regenerate efficiently (Kang and Lichtman 2013) after nervous injury, implicating aberrant signaling from sub‐synaptic myonuclei may contribute to reinnervation defects (Taetzsch and Valdez 2018) that lead to permanent denervation and atrophy. The mechanisms that drive defective transcription and translation of mitochondrial proteins in old age are multifactorial (Amorim et al. 2022), but one consequence of sub‐synaptic mitochondrial dysfunction at the NMJ is ineffective ATP production (Jia et al. 2007). Reduced ATP production from mitochondrial loss impacts synaptic transmission and synaptogenesis (Fu 1995), which is in line with our observations of defects in reinnervation kinetics. Furthermore, attenuation of the ability to produce ATP through oxidative phosphorylation contributes to reductions in energy needed for translation and protein folding at the endoplasmic reticulum (Brown and Naidoo 2012), which activates the mitochondrial unfolded protein response (Shpilka and Haynes 2018) and further increases reactive oxygen species production and oxidative DNA damage (Bohr et al. 2019). While changes in translation of different types of mitochondrial proteins at the NMJ require further investigation, our results suggest the loss in quantity of mitochondria predisposes neuromuscular synapses toward denervation in old age by impinging on the ability to reinnervate.

Key regulators of mitochondrial structure and function are nuclear‐encoded CHCH domain‐containing proteins, which are imported into the mitochondrial intermembrane and regulate folding into cristae and contact with the outermembrane. CHCH proteins regulate mitochondrial respiration, lipid composition, redox signaling, and membrane structure (Modjtahed et al. 2016), and mutations in CHCHD2 and CHCHD10 contribute to multiple types of neurodegenerative disorders (Jiang et al. 2022; Ikeda et al. 2022). However, CHCH‐proteins have not been studied in aging, and our results indicate the loss of CHCHD2 and CHCHD10 and a series of other mitochondrial proteins (Sun et al. 2016) contribute to several programs that render NMJs susceptible to degeneration during aging. First, the loss of CHCHD2 and CHCHD10 in aging may drive imbalances in iron homeostasis (Burstein et al. 2017), which has been shown to contribute to increases in membrane lipid peroxidation (Alves et al. 2021) and defects in proteostasis (Sayles et al. 2022). Next, CHCHD2 and CHCHD10 reside in the intermembrane space, which is rich in cholesterol that regulates contact between mitochondria and the endoplasmic reticulum (Marchi et al. 2014). The disruption of contact sites between the endoplasmic reticulum and mitochondria due to alterations in cholesterol metabolism has been shown to alter fusion/fission dynamics and insulin signaling (Rieusset 2018), which is in line with our data showing loss of CHCHD10 and CHCHD2 in sub‐synaptic myonuclei resulted in increased expression of *Retreg1*, which has been shown to drive ER fragmentation (Mookherjee et al. 2020) and autophagy (Castets et al. 2019) that develops from denervation. Summing these data shows that sub‐synaptic mitochondria and CHCH‐proteins are mediators of the nerve‐muscle interface, and their loss contributes to age‐related NMJ defects responsible for reinnervation.

Our findings here that sub‐synaptic mitochondria contribute to reinnervation and are lost with old age provide an important new understanding of NMJ health and axonal repair. Future work will focus on reactivation of several mitochondrial targets lost in aging and assessing whether these interventions can restore mitochondrial function and ability to reinnervate after injury.

## 4 Materials and Methods

### 4.1 Animals

All experimental procedures were approved by the University of Michigan Institutional Animal Care and Use Committee and conducted in accordance with National Institutes of Health guidelines. Animals were euthanized via intraperitoneal injection of tribromoethanol, followed by administration of a pneumothorax. Young (2–5 months) and old‐aged (23–29 months) C57BL/6J mice were used for all experiments involving sciatic nerve crush injury, mitochondrial analysis, and single‐nucleus RNA sequencing unless otherwise noted. For experiments involving AAV‐mediated genome editing, we utilized Acta1‐Cre; Rosa26^LSL‐Cas9‐EGFP^ mice, generated by crossing Acta1‐Cre (HSA‐Cre) transgenic mice with Rosa26^LSL‐Cas9‐EGFP mice^ (Jackson Laboratory strain #026179). This cross enabled skeletal muscle‐specific expression of Cas9 and EGFP following Cre‐mediated recombination. Acta1‐Cre; Rosa26^LSL‐Cas9‐EGFP^ mice were injected with AAV9 vectors carrying guide RNAs targeting CHCHD2 and CHCHD10 or with saline as a control. All mice were bred and maintained at the University of Michigan animal care facilities under standard housing conditions with ad libitum access to food and water and a 12‐h light/dark cycle. Both male and female mice were used in this study. Wherever possible, animals were assigned so that sexes were balanced within experimental groups; for experiments where exact balance was not attainable, the difference between sexes in any group was no greater than one animal. Because group sizes were limited, the study was not powered to detect sex‐specific effects, and results are reported on combined cohorts accordingly.

### 4.2 Sciatic Nerve Crush Injury

Mice were anesthetized with isoflurane and administered buprenorphine preoperatively. The surgical site was shaved, cleaned, and prepared under sterile conditions. A 3–4 mm incision was made parallel to the femur to expose the sciatic nerve, which was crushed for 30 s using hemostatic forceps locked at the third notch. The nerve was then repositioned, and the incision was closed with 6–0 prolene sutures. The contralateral leg served as an uninjured control. Mice were monitored postoperatively, and sutures were removed 7–10 days after surgery.

### 4.3 Immunofluorescent Staining

Tibialis anterior (TA) and gastrocnemius (GTN) muscles were collected at experimental endpoints, either cryosectioned longitudinally at 30 μm thickness or teased into muscle fiber bundles for whole‐mount staining, depending on the experiment. For cryosectioned samples, tissues were placed in 30% sucrose overnight, embedded in OCT, and flash‐frozen in isopentane chilled in liquid nitrogen. Sections were stored at −80°C until staining. For whole‐mount staining, TA muscles were fixed in 10% neutral‐buffered formalin for 15 min, washed in PBS, and mechanically dissected into bundles of 10–20 fibers.

All samples underwent blocking in normal goat serum and anti‐mouse Fab fragments, followed by incubation with primary antibodies targeting SV2 (DSHB, mouse IgG1, 1:50–1:100) and 2H3 (DSHB, mouse IgG1, 1:100–1:150) to label motor axon terminals and neurofilaments. Acetylcholine receptors (AChRs) were labeled with Alexa Fluor–conjugated α‐bungarotoxin (1:1000). Where applicable, additional markers included anti‐CHCHD10 (ProteinTech #25671‐1‐AP, 1:500) and anti–Cytochrome C (Cell Signaling #12963, 1:300). After washes, appropriate secondary antibodies conjugated to Alexa Fluor 405, 488, 594, or 647 (Invitrogen) were applied.

Sections and muscle fiber bundles were mounted with Fluoromount‐G or ProLong Diamond and imaged on either a Nikon A1R HD confocal or Zeiss LSM 900 Airyscan2 system. Z‐stacks were acquired at 40× with a 0.5 μm step size. Max intensity projections were generated in Fiji/ImageJ. NMJs were considered innervated if the presynaptic terminal (SV2/2H3) extensively overlapped with the postsynaptic AChR (BTX); otherwise, they were classified as denervated. Morphometric analysis of NMJs, including nerve terminal area, AChR area, overlap, fragmentation, and polyinnervation, was performed in Imaris 9.6 or Fiji as appropriate. For mitochondrial analysis, the Cytochrome C signal at the NMJ was binarized using the BTX‐defined NMJ region as a reference mask, and mitochondrial area and perimeter within this region were quantified.

### 4.4 Single‐Nucleus RNA‐Sequencing of Young and Aged Muscles Before and After Nerve Crush

Sciatic nerve crush injuries were performed as described above on young (2–3 months) and aged (> 23 months) mice. Nuclei were isolated for single‐nucleus RNA‐Sequencing (snRNA‐Seq) from injured and uninjured *tibialis anterior* (TA) muscles using one of two protocols: Dounce homogenization with Miltenyi Nuclei Extraction Buffer followed by optional CellPlex multiplexing or 10× Genomics Chromium Nuclei Isolation Kit.

#### 4.4.1 Dounce Homogenization With Miltenyi Nuclei Extraction Buffer

Nuclei extraction buffer was prepared by aliquoting 10 mL of Miltenyi Nuclei Extraction Buffer per muscle and adding Roche Protector RNase inhibitor to a final concentration of 0.2 U/μL and filter‐sterilizing. Resuspension buffer was prepared by dissolving bovine serum albumin (BSA) to a concentration of 0.1% (w/v) and Roche Protector RNase inhibitor to a final concentration of 0.2 U/μL in PBS and filter‐sterilizing. CellPlex wash buffer was prepared by dissolving 1% BSA and 0.2 U/μL of Roche Protector RNase inhibitor in PBS and filter‐sterilizing.

During the isolation procedure, all buffers, samples, and the Dounce homogenizer were kept on ice at all times to maintain nuclear integrity. TAs were harvested and placed into ice‐cold PBS until mincing. TAs were minced into very small pieces using surgical scissors until the muscle tissue appeared to be a homogeneous paste with no discernible chunks. The minced tissue was placed into the tube of a smooth glass 7 mL Dounce homogenizer along with 2 mL of nuclei extraction buffer. Pestle A was used to grind the minced tissue until a cloudy homogenate with few tissue chunks remained (approximately 10 strokes). The homogenate was transferred into a 50 mL conical tube, and any remaining homogenate in the Dounce homogenizer was collected by rinsing with an additional 2 mL of nuclei extraction buffer. Homogenates were diluted with an additional 5 mL of nuclei extraction buffer and further dissociated by pipetting with a 10 mL serological pipette. Samples were filtered through 70 μm and 40 μm cell strainers into new 50 mL conical tubes and centrifuged at 500*g* at 4°C for 5 min in a swinging bucket rotor centrifuge. The supernatant was aspirated, and nuclear pellets were resuspended in 5 mL of resuspension buffer and transferred to 15 mL conical tubes. The samples were centrifuged at 500*g* at 4°C for 5 min again. At this point, some samples were labeled with 10× Genomics CellPlex Cell Multiplexing Oligos (CMOs) following 10× Genomics CellPlex Protocol #1, and biological replicates were pooled before sequencing. Samples that weren't multiplexed were resuspended in 200–500 μL of resuspension buffer, and a 10 μL aliquot was mixed 1:1 with Trypan Blue to check nuclei number and quality with a hemocytometer.

#### 4.4.2 10× Genomics Chromium Nuclei Isolation

Lysis buffer, debris removal buffer, and wash/resuspension buffer were prepared according to the 10× Genomics Chromium Nuclei Isolation Kit User Guide: Chapter 1. TA muscles were harvested, rinsed in ice‐cold PBS, and cut into small cubes (1–2 mm). Muscle chunks were dabbed dry on a Kimwipe and transferred to a cryovial, which was then submerged in liquid nitrogen for 2–3 min to snap‐freeze the tissue. Nuclei were isolated from frozen tissue by following the 10× Genomics Chromium Nuclei Isolation Kit User Guide: Chapter 1 (with the slight modification of transferring samples to 15 mL conical tubes after the debris removal step, for ease of pelleting nuclei) and resuspended in 50 μL of wash/resuspension buffer before sequencing. Nuclei were checked for number and quality using a Luna FX7 automated fluorescent cell counter.

#### 4.4.3 Library Preparation and Sequencing

Libraries were prepared using the 10× Genomics Chromium Single Cell 3′ Standard or High‐Throughput kit version 3.1 and sequenced on an Illumina NovaSeq X using the 10B 300 cycle kit and a 2 × 150 bp run configuration, targeting 50,000 reads per cell.

### 4.5 snRNA‐Seq Data Processing and Analysis

FASTQ files were aligned to the GRCm39 mouse reference genome using Cell Ranger v8.0.0 (10× Genomics). Ambient RNA contamination was removed using DecontX (Gayoso et al. 2022), and only nuclei expressing more than 200 genes and with less than 10% mitochondrial read content were retained for downstream analysis. Putative doublets were removed using DoubletFinder (McGinnis et al. 2019). Scaling, normalization, variable feature selection, dimensionality reduction, and clustering were performed using Seurat with default parameters. Batch integration across samples was performed using scVI Tools (Lopez et al. 2018) and clustering was conducted with a resolution parameter set to 0.5. Cell type identities were assigned to clusters based on known marker genes. UMAP density plots were generated using the ggpointdensity package.

Pathway enrichment analysis was performed using the enrichGO() function in clusterProfiler, based on upregulated genes (log_2_FC > 1) identified with the FindMarkers() function in Seurat. Pathway enrichment scores were scaled as Z‐scores across groups for comparative analysis. Gene set enrichment analysis was performed using the gseGO() function in clusterProfiler (Xu et al. 2024). Mitochondrial module scoring was performed using curated gene sets derived from Gene Ontology (GO) terms and the Molecular Signatures Database (MSigDB); gene lists and module information are provided in Table 1.

Pseudotime trajectory analysis was performed on reclustered NMJ nuclei using FastMNN (Zhang et al. 2019) for batch correction, followed by diffusion map embedding with the Destiny package (Angerer et al. 2016). Lineage inference was conducted using Slingshot, with the trajectory root anchored in the NMJ subcluster enriched at Day 0. To identify genes with condition‐specific expression dynamics along pseudotime, we applied TradeSeq (Van den Berge et al. 2020) to fit generalized additive models (GAMs) to our pseudotime‐ordered nuclei. Pseudotime trajectories were inferred independently of treatment condition, and gene expression counts were modeled using fitGAM() with six knots. For condition testing, treatment groups (AAV and PBS) were initially assessed separately and then collapsed into two categories (“AAV” vs. “PBS”) for differential expression analysis using the conditionTest() function. *p*‐values were adjusted using the Benjamini–Hochberg method to control the false discovery rate (FDR), and genes with adjusted *p*‐values < 0.05 were considered significant.

### 4.6 Mitochondrial Isolation and Seahorse XF Assay

Mitochondria were isolated from tibialis anterior (TA) muscles using the Miltenyi Biotec Mitochondrial Isolation Kit for mouse tissue according to the manufacturer's instructions. Tissues were homogenized, magnetically labeled with anti‐TOM22 microbeads, and purified by magnetic separation. Isolated mitochondria were quantified using the Qubit Protein Assay Kit and kept on ice until use.

Mitochondrial bioenergetic function was assessed using the Seahorse XF96 Analyzer (Agilent) following a modified standard protocol. Briefly, mitochondria were plated onto Seahorse XF96 cell culture plates and centrifuged to adhere. Assays were performed in mitochondrial assay solution containing malate and glutamate as substrates. Oxygen consumption rate (OCR) measurements were collected at baseline and after sequential injections of ADP, oligomycin, FCCP, and rotenone. Data were analyzed using Wave software (Agilent), and mitochondrial parameters, including ATP production, proton leak, maximal respiration, and respiratory control ratio, were calculated.

#### 4.6.1 Western Blotting

Tibialis anterior (TA) muscles were harvested and homogenized to extract total protein lysates. Protein concentrations were determined using a BCA assay, and 20 μg of protein per sample was loaded onto 12% SDS‐polyacrylamide gels for electrophoresis. Proteins were transferred to PVDF membranes, blocked, and incubated overnight at 4°C with primary antibodies against CHCHD2 (Proteintech, 663021‐Ig) and CHCHD10 (Proteintech, 25671‐1‐AP). After washing, membranes were incubated with horseradish peroxidase (HRP)‐conjugated secondary antibodies and developed using chemiluminescence detection. Protein levels were normalized to GAPDH (Cell Signaling Technologies, 2118S) as a loading control.

#### 4.6.2 In Vivo CRISPR‐Cas9 Gene Knockout of Chchd2 and Chchd10 in Myonuclei

AAVs targeting *CHCHD10* and *CHCHD2* were manufactured by VectorBuilder. Briefly, gRNAs targeting *CHCHD10* (ACCGCCGCAGGCGTAGCCGT) and *CHCHD2* (CCCCGAGGGTCTCACCTGGC), followed by scaffold sequences specific to *S. pyogenes* Cas9 (GTTTTAGAGCTAGAAATAGCAAGTTAAAATAAGGCTAGTCCGTTATCAACTTGAAAAAGTGGCACCGAGTCGGTGC), were inserted into a pAAV plasmid backbone under U6 promoters. An mCherry reporter was included under a CAG promoter to facilitate easy visualization of virus transduction, and an ampicillin resistance gene was included to allow for selection of transformed *E. coli* . The plasmid was assembled via Gibson cloning, validated via restriction enzyme digestion, and transformed into VB UltraStable *E. coli* for expansion. Plasmid DNA was isolated from transformed *E. coli* and packaged into AAV9 viral particles by VectorBuilder for downstream use.

AAVs were thawed on ice and resuspended in sterile PBS at a concentration of 5e9 vg/μL. AAVs were injected intramuscularly in a volume of either 20 μL (1e11 vg) into the tibialis anterior (TA) or 30 μL (1.5e11 vg) into the gastrocnemius muscle. To perform intramuscular injections, mice were anesthetized via isoflurane inhalation, and their hindlimbs were shaved to remove fur from the injection site. The injection site was wiped with 70% ethanol, and injections were drawn up into BD Lo‐Dose U‐100 insulin syringes (with 0.5 mL volume and 28G needle). Muscles were either injected with the AAV suspension or an equal volume of PBS vehicle control. Mice were allowed to recover for at least 3 weeks before undergoing nerve crush surgery or tissue isolation.

#### 4.6.3 qPCR Validation Following CHCHD2/CHCHD10 Knockout in ACTA1‐Cas9 Mice

Three young (3 months) male ACTA1‐Cas9 mice were intramuscularly injected with AAV suspension or PBS controls in the TA muscles as described above and allowed to recover for 3 weeks before harvesting TAs and embedding in OCT for RNA isolation from cryosections, cDNA synthesis, and qPCR as described in the previous section. *Chchd2* and *Chchd10* primers were purchased from IDT (Table 2) and used at a 500 nM working primer concentration. Gene expression was normalized to the *Gapdh* housekeeping gene and control tissues using the ∆∆Ct method.

**TABLE 2:** Primer sequences for RT‐qPCR.

Gene Name	IDT assay number	Forward sequence (5′‐3′)	Reverse sequence (5′‐3′)
*Gapdh*	Mm.PT.39a.1	AATGGTGAAGGTCGGTGTG	GTGGAGTCATACTGGAACATTAG
*Chchd2*	Mm.PT.58.5102404	CTGGACGCTTCTGTACGTTT	GGAGCAGCCCTCATCTGA
*Chchd10*	Mm.PT.58.7935852	CTCAACTCCAGCCTTATGCG	CACCAAAGCCACATAACATTCC

#### 4.6.4 Transmission Electron Microscopy Imaging of Neuromuscular Junctions

Transmission electron micrographs were collected of mouse neuromuscular junctions as described previously (Hastings et al. 2023). In brief, one young (3‐month‐old) male mouse and one old‐aged (29‐month‐old) male mouse were perfused transcardially with sodium cacodylate buffer followed by 2% PFA/3% glutaraldehyde. Extensor digitorum longus (EDL) muscles were dissected in buffer, and the largest EDL compartment was fixed overnight in 2% PFA/3% glutaraldehyde. The EDL compartment was then cut in half perpendicular to its length with a razor blade near the endplate band. These muscle pieces were then stained in 1% osmium tetroxide, 1% ferrocyanide for 5 h, followed by staining with 1% uranyl acetate for 2 h. Muscles were dehydrated in graded ethanol and then embedded in SPURR Low Viscosity Embedding Kit, Hard Mix. A Leica EM UC7 Ultramicrotome with a diamond knife was used to trim the blocks and to obtain more than 40 individual 100 nm cross sections per sample, which were mounted on bare 200 mesh or 300 mesh copper grids. The grids were imaged under a Philips EM410 Transmission Electron Microscope with a NANOSPRT5 camera and AMT V701 software. Digital images of individual NMJs were captured from sections at magnifications between 12,000× and 21,000×. 13 images were taken of young adult NMJs, and 9 images were taken of old‐aged NMJs. It was not possible to determine which of these images were of different NMJs or whether the images were of the same NMJ at different depths. Adobe Photoshop 2022 (version 23.2) was used to highlight mitochondria in red.

Transmission electron micrographs were analyzed in ImageJ. Mitochondria density was calculated for muscle postsynaptic regions in which these structures were easily identifiable by dividing counts of mitochondria within an arch‐shaped region emanating 1.5 μm from the synaptic cleft into the muscle by the area of this region, which was measured using the polygon tool in ImageJ. A total of 17 synaptic regions were analyzed from young NMJs, and 14 synaptic regions were analyzed from old‐aged NMJs.

## Author Contributions

S.D.G., P.M.F., K.I., E.K.F., D.J., G.K., G.V., and J.V.C. performed experiments. S.D.G., P.F., and G.K. analyzed data. S.D.G., P.F., and C.A.A. designed the experiments. S.D.G., P.F., and C.A.A. wrote and edited the manuscript with assistance from other authors.

## Funding

This work was supported by Congressionally Directed Medical Research Programs, W81XWH2010336, W81XWH2110491; National Science Foundation, 2045977; Hevolution Foundation, HF‐AGE 001; and Defense Advanced Research Projects Agency, D20AC0002.

## Conflicts of Interest

The authors declare no conflicts of interest.

## Supporting information


**Figure S1:** Age‐associated differences in NMJ morphology and reinnervation kinetics. (A) Quantification of mitochondrial perimeter and NMJ morphological parameters in tibialis anterior muscles from young and old‐aged mice. Measurements include AChR perimeter, endplate area, endplate perimeter, endplate diameter, and compactness (*n* = 3–4 mice per group). (B) Seahorse mitochondrial flux assay comparing mitochondria isolated from young and aged GTN muscles. (C) ATP production (pmol/min) and (D) maximal respiration measurements derived from the mitochondrial flux assay shown in (B). (E) Representative images of NMJs stained for presynaptic markers (NF/SV2, red), postsynaptic AChRs (BTX, green), and nuclei (DAPI, blue) from young and old‐aged mice at baseline (sham) and one week following sciatic nerve crush. Scale bars, 10 μm. (F) Quantification of the percentage of denervated NMJs at 1, 2, and 8 weeks post‐injury in young and aged muscles (*n* = 3 mice per time point, ANOVA, Šidák's multiple comparisons test, ****p < 0.0001, **p < 0.01).
**Figure S2:** Single‐nucleus RNA‐seq quality control, cell type classification, and pathway enrichment across myonuclei. (A) Violin plots showing the number of detected genes, UMIs, and percentage of mitochondrial reads per nucleus across young and aged samples at Day 0, Day 7, and Day 14 post‐sciatic nerve crush. (B) Dot plot displaying expression levels and detection frequencies of selected marker genes used for cell type identification. Dot size indicates the percentage of nuclei expressing the gene; color intensity reflects average expression. (C) Stacked bar plots showing relative proportions of annotated myonuclei (top) and non‐myonuclei (bottom) cell types across timepoints and age groups. (D) Heatmap showing Z‐scored enrichment of biological pathways in individual myonuclear clusters, including oxidative phosphorylation, extracellular matrix organization, and striated muscle development. (E) Dot plot showing GO term enrichment for Type IIx/IIb myonuclei. Dot size reflects the number of associated genes; color indicates adjusted *p*‐value. (F–G) Volcano plots displaying differentially expressed genes in Igfn1^+^ (F) and Ncam1^+^ (G) myonuclei comparing young and aged muscles at 14 days post‐injury.
**Figure S3:** NMJ myonuclear subcluster analysis reveals enrichment of mitochondrial‐related pathways and age‐associated transcriptional differences. (A) Heatmap showing Z‐scored enrichment of cellular component GO terms across reclustered NMJ myonuclear subclusters. Each subcluster is annotated by enriched pathways, including neuromuscular junction, postsynaptic specialization, mitochondrial complexes, and cytoskeletal components. (B) Gene Set Enrichment Analysis (GSEA) comparing NMJ nuclei from young vs. aged muscles at 14 days post‐injury shows significant enrichment for mitochondrial pathways—including inner mitochondrial membrane protein complex, respirasome, and electron transport chain components—in young muscle. (C) Violin plots of module scores for mitochondrial biogenesis, degradation, oxidative phosphorylation (OXPHOS), and protein import across NMJ nuclei from young and aged muscles at Day 0, Day 7, and Day 14 post‐injury. (D) Violin plots of expression for *Chchd10*, *Chchd2*, and *Cycs* across NMJ nuclei and timepoints. Statistical significance determined by Wilcoxon test; *p* < 0.05, *p* < 0.01, *p* < 0.001.
**Figure S4:** AAV delivery results in efficient expression of mCherry in skeletal muscle. (A, B) Schematic of gRNA target sites within exon 1/intron 1 of CHCHD2 (A) and exon 3 of CHCHD10 (B), showing guide RNA (blue), PAM site (magenta), and Cas9 cleavage position. gRNAs were designed to target the negative strand, and cut sites are indicated by blue triangles. Genomic coordinates are based on the mm10 assembly. (C) Representative muscle fiber bundles of tibialis anterior muscles from Acta1‐Cre; Rosa26^LSL‐Cas9‐eGFP^ mice injected with either saline (–AAV) or AAV9 encoding mCherry and CHCHD2/CHCHD10‐targeting guide RNAs (+AAV). GFP (green) indicates Cas9 expression; mCherry (red) marks AAV‐transduced fibers. Scale bar = 100 μm.
**Figure S5:** Quantification of neuromuscular junction morphology metrics following sciatic nerve crush with or without CHCHD2/CHCHD10 knockout. Line graphs showing temporal changes in NMJ morphological parameters at Day 0, Day 14, and Day 28 post‐injury in tibialis anterior muscles injected with either saline (–AAV) or CHCHD2/CHCHD10‐targeting AAV (+AAV). Metrics include nerve terminal area, nerve terminal perimeter, AChR perimeter, endplate perimeter, endplate diameter, overlap between pre‐ and postsynaptic compartments, synaptic area, percentage of denervated NMJs, and number of terminal blebs. Data represent mean ± SEM; *n* = 3–4 mice per group.
**Figure S6:** Single‐nucleus RNA‐seq quality control and cell type annotation in muscles with and without CHCHD2/CHCHD10 knockout. (A) Violin plots showing distribution of gene counts, UMI counts, and percentage of mitochondrial reads per nucleus across four experimental groups: –AAV Day 0, –AAV Day 14, +AAV Day 0, and + AAV Day 14. (B) Dot plot of selected marker genes used to annotate major cell populations. Dot size represents the percentage of nuclei expressing each gene; color indicates average expression. (C) Stacked bar plots of annotated myonuclear (top) and non‐myonuclear (bottom) populations across all conditions. (D) Expression of canonical NMJ‐related genes (*Gdnf*, *Colq*, *Kcnq5*) across pseudotime in –AAV and +AAV conditions. (E) Expression of stress‐ and translation‐related genes (*Cryab*, *Igfbp5*, *Retreg1*) across pseudotime.

## Data Availability

The data that support the findings of this study are openly available in GEO at https://www.ncbi.xyz/geo/browse/?view=samples&series=296807, reference number GSE296807.
