# Supplementary material for: Age‐Associated Dysregulation of Postsynaptic Mitochondria Perturbs Reinnervation Kinetics
Source: Aging Cell. 2026 Jan 6;25(1):e70355. doi: 10.1111/acel.70355 (PMC12775679; doi:10.1111/acel.70355)
Supplement: Supplementary file 1 — Figure S1: Age‐associated differences in NMJ morphology and reinnervation kinetics. (A) Quantification of mitochondrial perimeter and NMJ morphological parameters in tibialis anterior muscles from young and old‐aged mice. Measurements include AChR perimeter, endplate area, endplate perimeter, endplate diameter, and compactness (n = 3–4 mice per group). (B) Seahorse mitochondrial flux assay comparing mitochondria isolated from young and aged GTN muscles. (C) ATP production (pmol/min) and (D) maximal respiration measurements derived from the mitochondrial flux assay shown in (B). (E) Representative images of NMJs stained for presynaptic markers (NF/SV2, red), postsynaptic AChRs (BTX, green), and nuclei (DAPI, blue) from young and old‐aged mice at baseline (sham) and one week following sciatic nerve crush. Scale bars, 10 μm. (F) Quantification of the percentage of denervated NMJs at 1, 2, and 8 weeks post‐injury in young and aged muscles (n = 3 mice per time point, ANOVA, Šidák's multiple comparisons test, ****p < 0.0001, **p < 0.01). Figure S2: Single‐nucleus RNA‐seq quality control, cell type classification, and pathway enrichment across myonuclei. (A) Violin plots showing the number of detected genes, UMIs, and percentage of mitochondrial reads per nucleus across young and aged samples at Day 0, Day 7, and Day 14 post‐sciatic nerve crush. (B) Dot plot displaying expression levels and detection frequencies of selected marker genes used for cell type identification. Dot size indicates the percentage of nuclei expressing the gene; color intensity reflects average expression. (C) Stacked bar plots showing relative proportions of annotated myonuclei (top) and non‐myonuclei (bottom) cell types across timepoints and age groups. (D) Heatmap showing Z‐scored enrichment of biological pathways in individual myonuclear clusters, including oxidative phosphorylation, extracellular matrix organization, and striated muscle development. (E) Dot plot showing GO term enrichment for Ty [file ACEL-25-e70355-s001.pdf]

Figure S1

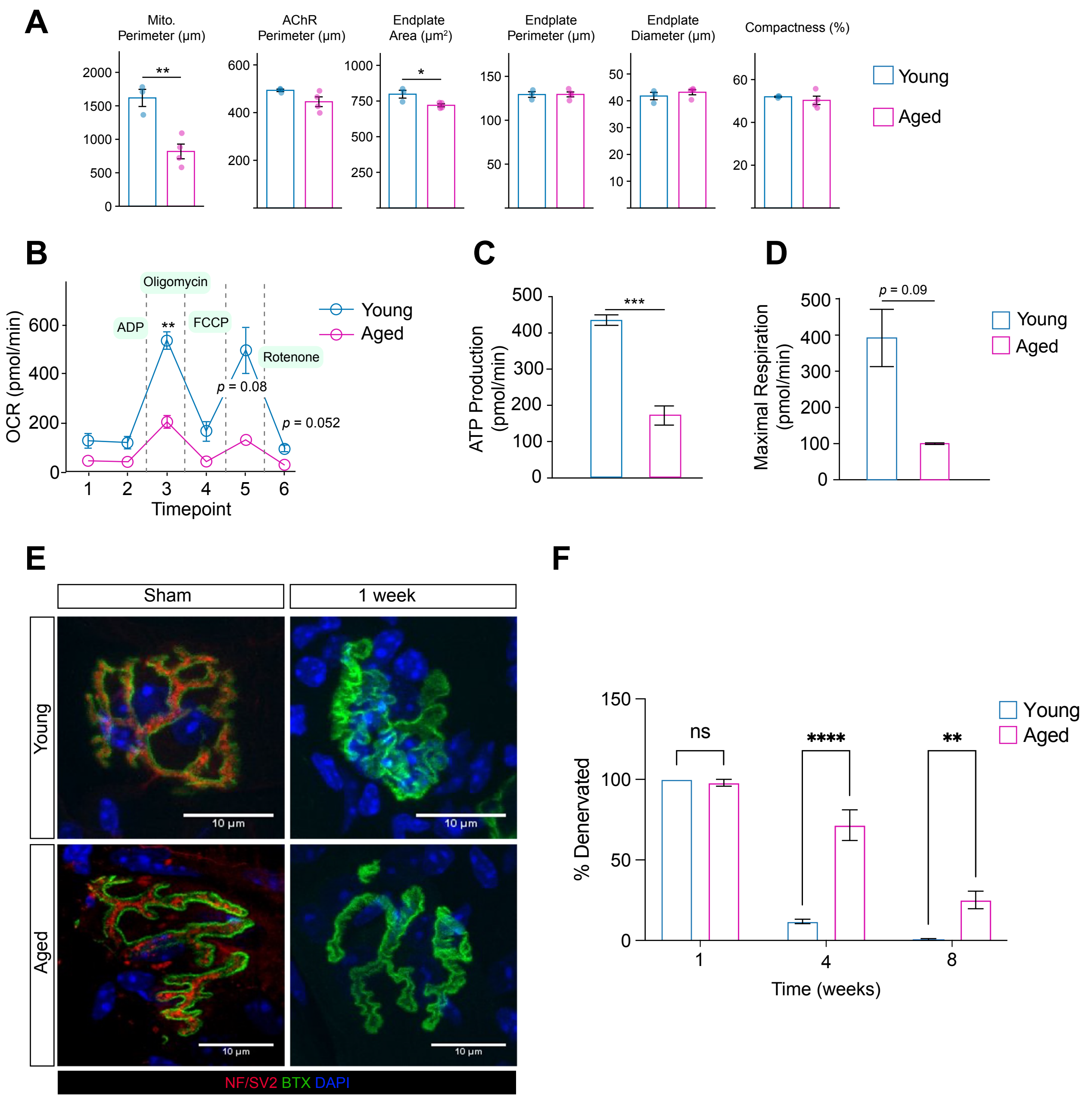
A
Mito. Perimeter (µm)
AChR Perimeter (µm)
Endplate Area (µm²)
Endplate Perimeter (µm)
Endplate Diameter (µm)
Compactness (%)
Young
Aged
B
OCR (pmol/min)
Timepoint
Oligomycin
ADP
FCCP
Rotenone
p = 0.08
p = 0.052
C
ATP Production (pmol/min)
D
Maximal Respiration (pmol/min)
p = 0.09
E
Sham
1 week
Young
Aged
10 µm
NF/SV2 BTX DAPI
F
% Denervated
Time (weeks)
ns

Figure S2

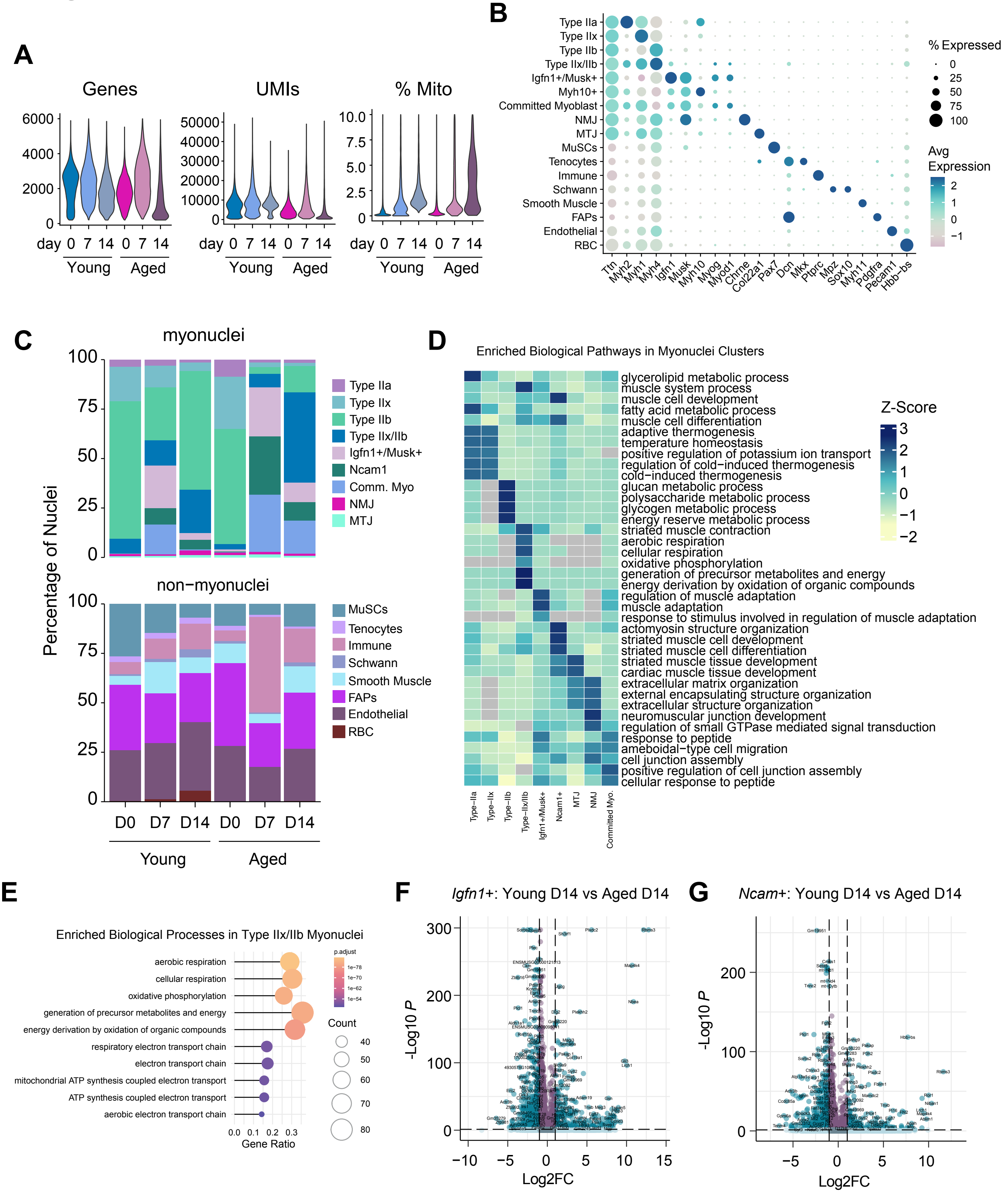
A
Genes
UMIs
% Mito
day 0 7 14 0 7 14
Young
Aged
B
Type IIa
Type IIx
Type IIb
Type IIx/IIb
Igfn1+/Musk+
Myh10+
Committed Myoblast
NMJ
MTJ
MuSCs
Tenocytes
Immune
Schwann
Smooth Muscle
FAPs
Endothelial
RBC
Ttn Myh2 Myh1 Myh4 Igfn1 Musk Myh10 Myog Myod1 Chrne Col22a1 Pax7 Dcn Mkx Ptprc Mpz Sox10 Myh11 Pdgfra Pecam1 Hbb-bs
% Expressed
Avg Expression
C
myonuclei
non−myonuclei
Percentage of Nuclei
Type IIa
Type IIx
Type IIb
Type IIx/IIb
Igfn1+/Musk+
Ncam1
Comm. Myo
NMJ
MTJ
MuSCs
Tenocytes
Immune
Schwann
Smooth Muscle
FAPs
Endothelial
RBC
D0 D7 D14 D0 D7 D14
Young
Aged
D
Enriched Biological Pathways in Myonuclei Clusters
glycerolipid metabolic process
muscle system process
muscle cell development
fatty acid metabolic process
muscle cell differentiation
adaptive thermogenesis
temperature homeostasis
positive regulation of potassium ion transport
regulation of cold−induced thermogenesis
cold−induced thermogenesis
glucan metabolic process
polysaccharide metabolic process
glycogen metabolic process
energy reserve metabolic process
striated muscle contraction
aerobic respiration
cellular respiration
oxidative phosphorylation
generation of precursor metabolites and energy
energy derivation by oxidation of organic compounds
regulation of muscle adaptation
muscle adaptation
response to stimulus involved in regulation of muscle adaptation
actomyosin structure organization
striated muscle cell development
striated muscle cell differentiation
striated muscle tissue development
cardiac muscle tissue development
extracellular matrix organization
external encapsulating structure organization
extracellular structure organization
neuromuscular junction development
regulation of small GTPase mediated signal transduction
response to peptide
ameboidal−type cell migration
cell junction assembly
positive regulation of cell junction assembly
cellular response to peptide
Z-Score
Type−IIa Type−IIx Type−IIb Type−IIx/IIb Igfn1+/Musk+ Ncam1+ MTJ NMJ Committed Myo.
E
Enriched Biological Processes in Type IIx/IIb Myonuclei
aerobic respiration
cellular respiration
oxidative phosphorylation
generation of precursor metabolites and energy
energy derivation by oxidation of organic compounds
respiratory electron transport chain
electron transport chain
mitochondrial ATP synthesis coupled electron transport
ATP synthesis coupled electron transport
aerobic electron transport chain
p.adjust
Count
Gene Ratio
F
Igfn1+: Young D14 vs Aged D14
-Log10 P
Log2FC
G
Ncam+: Young D14 vs Aged D14
-Log10 P
Log2FC

Figure S3

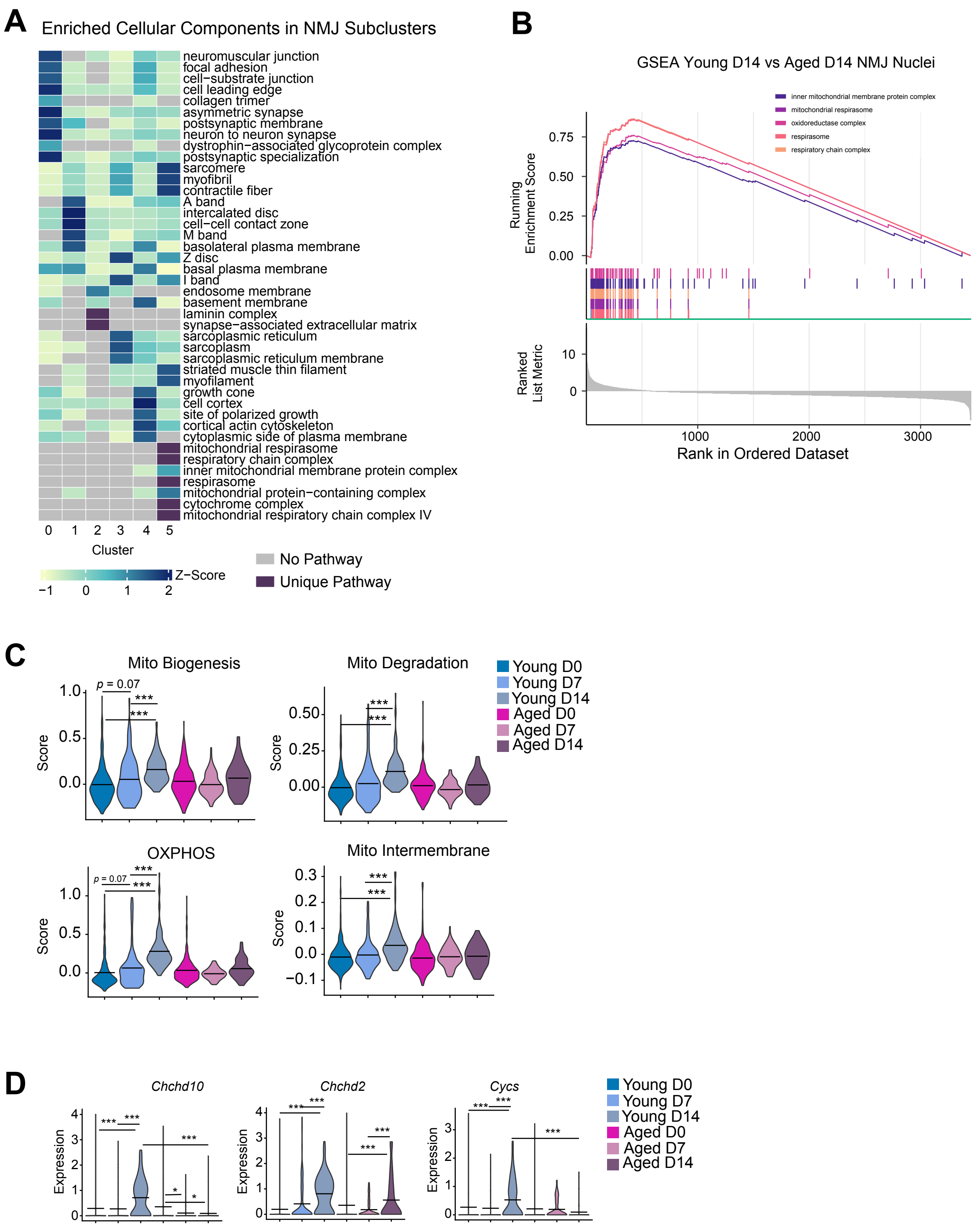

A
Enriched Cellular Components in NMJ Subclusters
neuromuscular junction
focal adhesion
cell−substrate junction
cell leading edge
collagen trimer
asymmetric synapse
postsynaptic membrane
neuron to neuron synapse
dystrophin−associated glycoprotein complex
postsynaptic specialization
sarcomere
myofibril
contractile fiber
A band
intercalated disc
cell−cell contact zone
M band
basolateral plasma membrane
Z disc
basal plasma membrane
I band
endosome membrane
basement membrane
laminin complex
synapse−associated extracellular matrix
sarcoplasmic reticulum
sarcoplasm
sarcoplasmic reticulum membrane
striated muscle thin filament
myofilament
growth cone
cell cortex
site of polarized growth
cortical actin cytoskeleton
cytoplasmic side of plasma membrane
mitochondrial respirasome
respiratory chain complex
inner mitochondrial membrane protein complex
respirasome
mitochondrial protein−containing complex
cytochrome complex
mitochondrial respiratory chain complex IV
0 1 2 3 4 5
Cluster
Z−Score
−1 0 1 2
No Pathway
Unique Pathway
B
GSEA Young D14 vs Aged D14 NMJ Nuclei
inner mitochondrial membrane protein complex
mitochondrial respirasome
oxidoreductase complex
respirasome
respiratory chain complex
Running Enrichment Score
0.75
0.50
0.25
0.00
Ranked List Metric
10
0
1000
2000
3000
Rank in Ordered Dataset
C
Mito Biogenesis
Mito Degradation
OXPHOS
Mito Intermembrane
Score
p = 0.07
***
Young D0
Young D7
Young D14
Aged D0
Aged D7
Aged D14
D
Chchd10
Chchd2
Cycs
Expression
***
*

Figure S4

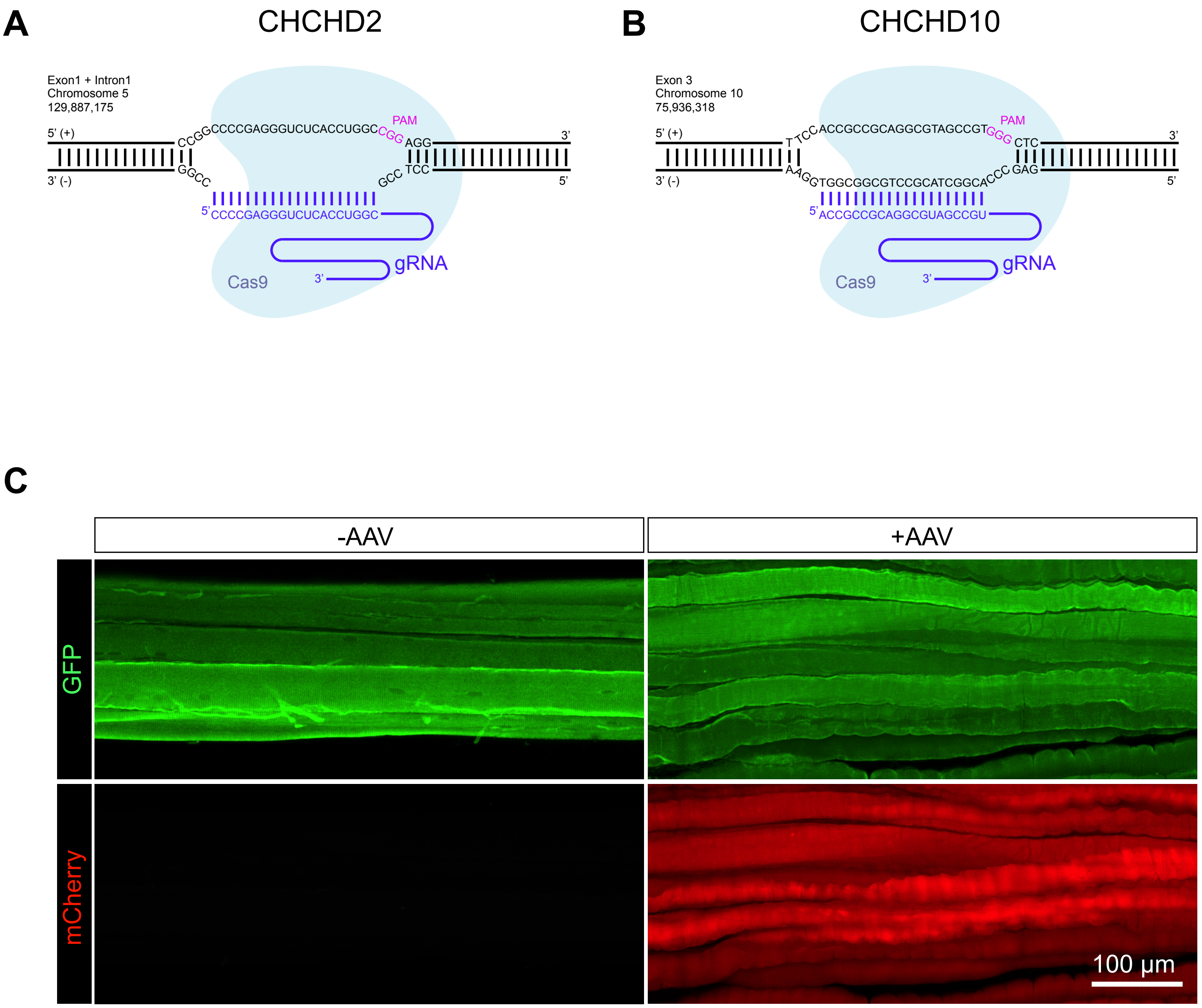
A
CHCHD2
Exon1 + Intron1
Chromosome 5
129,887,175
5' (+)
3' (-)
CCGGCCCCGAGGGUCUCACCUGGCCGG
PAM
AGG
TCC
GGCC
GCC
3'
5'
5' CCCCGAGGGUCUCACCUGGC
gRNA
Cas9
B
CHCHD10
Exon 3
Chromosome 10
75,936,318
TTCCACCGCCGCAGGCGTAGCCGTGGG
PAM
CTC
GAG
AAGGTGGCGGCGTCCGCATCGGCACCC
5' ACCGCCGCAGGCGUAGCCGU
gRNA
Cas9
C
-AAV
+AAV
GFP
mCherry
100 μm

Figure S5

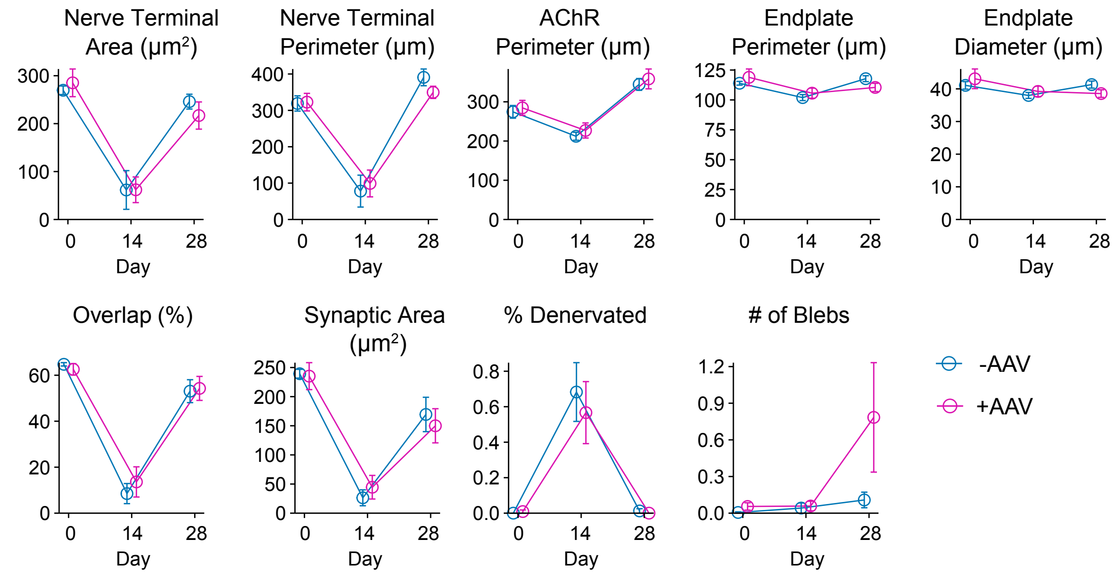
Nerve Terminal Area (µm²)
Nerve Terminal Perimeter (µm)
AChR Perimeter (µm)
Endplate Perimeter (µm)
Endplate Diameter (µm)
Overlap (%)
Synaptic Area (µm²)
% Denervated
# of Blebs
Day
0
14
28
-AAV
+AAV

Figure S6

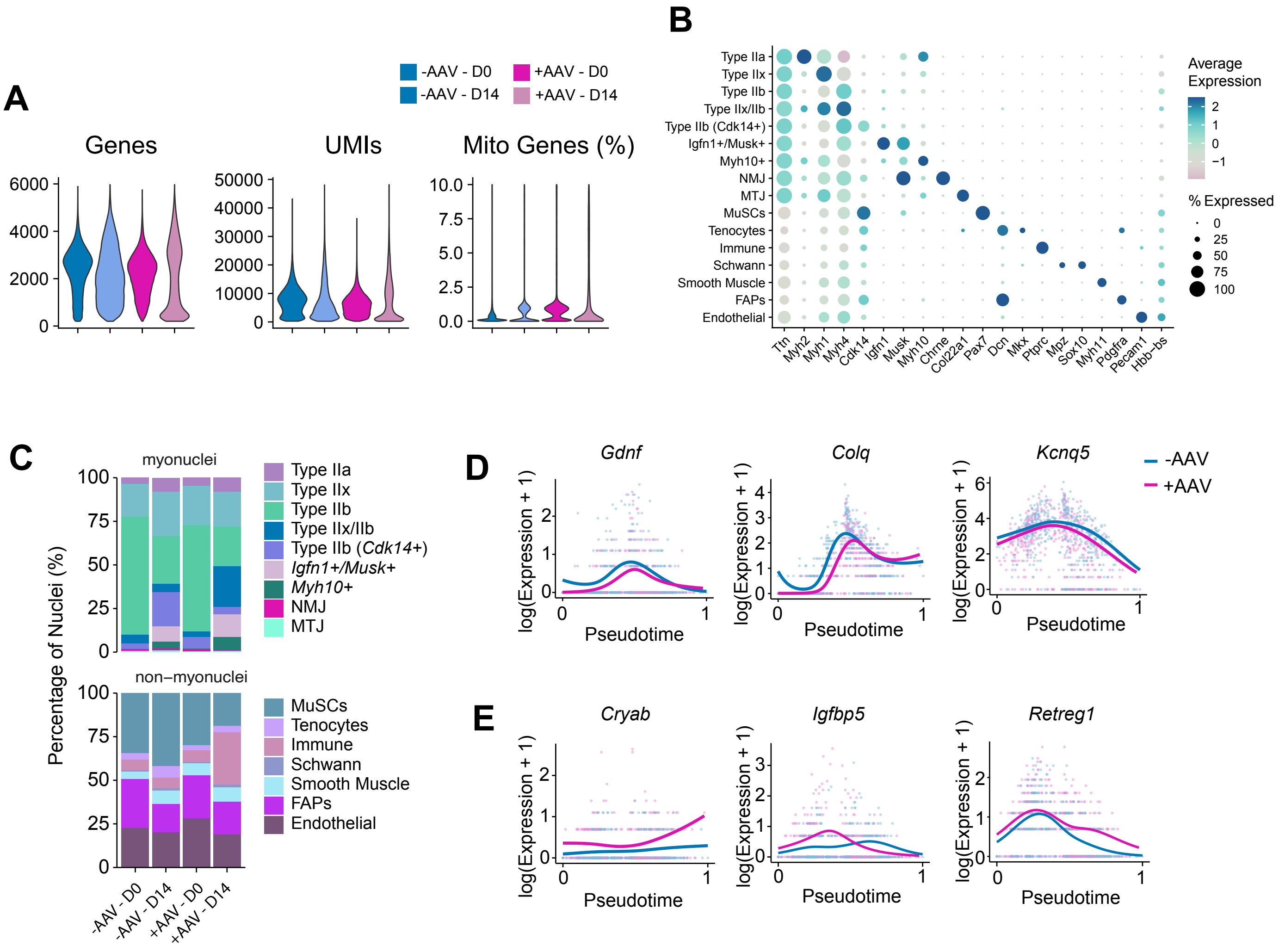

A
-AAV - D0
+AAV - D0
-AAV - D14
+AAV - D14
Genes
UMIs
Mito Genes (%)
B
Type IIa
Type IIx
Type IIb
Type IIx/IIb
Type IIb (Cdk14+)
Igfn1+/Musk+
Myh10+
NMJ
MTJ
MuSCs
Tenocytes
Immune
Schwann
Smooth Muscle
FAPs
Endothelial
Ttn
Myh2
Myh1
Myh4
Cdk14
Igfn1
Musk
Myh10
Chrne
Col22a1
Pax7
Dcn
Mkx
Ptprc
Mpz
Sox10
Myh11
Pdgfra
Pecam1
Hbb-bs
Average Expression
% Expressed
C
myonuclei
non–myonuclei
Percentage of Nuclei (%)
Type IIa
Type IIx
Type IIb
Type IIx/IIb
Type IIb (Cdk14+)
Igfn1+/Musk+
Myh10+
NMJ
MTJ
MuSCs
Tenocytes
Immune
Schwann
Smooth Muscle
FAPs
Endothelial
-AAV - D0
-AAV - D14
+AAV - D0
+AAV - D14
D
Gdnf
Colq
Kcnq5
-AAV
+AAV
log(Expression + 1)
Pseudotime
E
Cryab
Igfbp5
Retreg1
log(Expression + 1)
Pseudotime
